# Glutathione‐Scavenging Nanoparticle‐Mediated PROTACs Delivery for Targeted Protein Degradation and Amplified Antitumor Effects

**DOI:** 10.1002/advs.202207439

**Published:** 2023-04-17

**Authors:** Hai‐Jun Liu, Wei Chen, Gongwei Wu, Jun Zhou, Chuang Liu, Zhongmin Tang, Xiangang Huang, Jingjing Gao, Yufen Xiao, Na Kong, Nitin Joshi, Yihai Cao, Reza Abdi, Wei Tao

**Affiliations:** ^1^ Center for Nanomedicine and Department of Anesthesiology Brigham and Women's Hospital Harvard Medical School Boston MA 02115 USA; ^2^ Transplantation Research Center Renal Division Brigham and Women's Hospital Harvard Medical School Boston MA 02115 USA; ^3^ Department of Medical Oncology Dana‐Farber Cancer Institute Harvard Medical School Boston MA 02215 USA; ^4^ Department of Microbiology Tumor and Cell Biology Karolinska Institute Stockholm 171 77 Sweden

**Keywords:** BRD4, c‐Myc, cancer therapy, glutathione scavenging, PROteolysis TArgeting Chimeras nanoparticles

## Abstract

PROteolysis TArgeting Chimeras (PROTACs) are an emerging class of promising therapeutic modalities that selectively degrade intracellular proteins of interest by hijacking the ubiquitin‐proteasome system. However, the lack of techniques to efficiently transport these degraders to targeted cells and consequently the potential toxicity of PROTACs limit their clinical applications. Here, a strategy of nanoengineered PROTACs, that is, Nano‐PROTACs, is reported, which improves the bioavailability of PROTACs and maximizes their capacity to therapeutically degrade intracellular oncogenic proteins for tumor therapy. The Nano‐PROTACs are developed by encapsulating PROTACs in glutathione (GSH)‐responsive poly(disulfide amide) polymeric (PDSA) nanoparticles and show that ARV@PDSA Nano‐PROTAC, nanoengineered BRD4 degrader ARV‐771, improves BRD4 protein degradation and decreases the downstream oncogene c‐Myc expression. Benefiting from the GSH‐scavenging ability to amply the c‐Myc‐related ferroptosis and cell cycle arrest, this ARV@PDSA Nano‐PROTACs strategy shows superior anti‐tumor efficacy with a low dose administration and good biocompatibility in vivo. The findings reveal the potential of the Nano‐PROTACs strategy to treat a broad range of diseases by dismantling associated pathogenic proteins.

## Graphical abstract

Nanoengineered PROteolysis TArgeting Chimeras (Nano‐PROTACs) are developed with glutathione (GSH)‐responsive polymeric nanoparticles and pioneer their applications in cancer therapy. The Nano‐PROTACs not only address the poor druggability of PROTACs but also enhance the therapeutic efficacy via an intelligent GSH‐scavenging strategy to amplify  c‐Myc‐related ferroptosis and cell cycle arrest. This GSH‐scavenging PROTAC nanotechnology would be generalized for treating different malignancies in vivo.

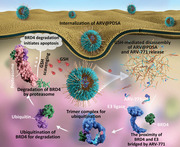

## 1 Introduction

PROteolysis TArgeting Chimeras (PROTACs) to hijack the ubiquitin‐proteasome system (UPS) and degrade targeted proteins is an emerging therapeutic strategy, noted for its high selectivity and efficiency of protein degradation.^[^ ^1^, ^2^ ^]^ PROTACs are heterobifunctional small molecules consisting of two ligands joined by a linker: one ligand recruits and binds the targeted proteins and the other recruits and binds an E3 ubiquitin ligase,^[^ ^3^, ^4^, ^5^ ^]^ thus leading to the ubiquitylation of the targeted proteins followed by their degradation by UPS. This unique therapeutic technique offers several unprecedented advantages including i) expanding the therapeutic landscape to encompass undruggable proteins that have historically been considered highly challenging to target with small‐molecule inhibitors, and ii) acting on the targeted proteins in a catalyst‐like manner, which can significantly reduce systemic exposure to the administered molecules.^[^ ^3^, ^6^ ^]^

Recently, two first‐in‐human clinical trials (NCT03888612 and NCT04072952) reported promising therapeutic outcomes with orally delivered ARV‐110 and ARV‐471 PROTACs for patients with metastatic castration‐resistant prostate cancer and metastatic breast cancer, respectively.^[^ ^7^, ^8^ ^]^ However, despite such positive results, the physicochemical properties of most PROTACs still pose great challenges for translational research, and systemic (i.e., intravenous) administration of PROTACs in the clinic remains elusive for several reasons. First, their large molecular weight (>800 Da) and the presence of multiple hydrogen bond donors and acceptors of PROTACs lead to poor cell membrane permeability, significantly limiting their intracellular availability.^[^ ^2^, ^9^ ^]^ Second, their hydrophobic nature makes water‐insoluble PROTACs likely to aggregate in physiological environments, reducing their accumulation in targeted cells. To address these obstacles, recent years have seen the design of medium‐assisted strategies to improve therapeutic outcomes with PROTACs.^[^ ^10^ ^]^ Although these strategies have substantially increased solubility for in vivo studies, remaining challenges include their short in vivo half‐life, rapid renal clearance, insufficient accumulation in lesion areas, and potential toxicity of high doses of PROTACs.^[^ ^5^, ^11^, ^12^ ^]^ Moreover, despite numerous efforts to improve the therapeutic efficacy and minimize the side effects of PROTACs by chemically conjugating them with antibodies, aptamers, folate, or peptides,^[^ ^13^, ^14^, ^15^ ^]^ translating these chemically synthesized PROTACs into clinical use is severely limited by technical challenges, high manufacturing costs, and low effectiveness resulting from such complicated synthetic processes.

Nanotechnology has the unique advantage of substantially improving the bioavailability of encapsulated active pharmaceutical ingredients;^[^ ^16^, ^17^, ^18^, ^19^, ^20^, ^21^ ^]^ recently, attempts have been made to design nanoplatforms to enhance the bioavailability of PROTACs for cancer treatment.^[^ ^22^, ^23^, ^24^ ^]^ However, most of these studies neither provided in vivo evidence of enhanced targeted protein degradation nor did they demonstrate that the nanoparticle‐assisted PROTACs strategy improves therapeutic outcomes in preclinical studies. A few recent studies attempted to integrate PROTACs molecules onto the backbone of amphiphilic polymers via cleavable covalent bonds leveraging tumor intrinsic biomarkers. Along with the polymers assembling into nanoparticles, these hitched PROTACs molecules were easily hitchhiked on the nanoparticles, followed by get‐off in virtue of the tumor biomarkers such as intercellular highly expressed GSH^[^ ^25^ ^]^ and cathepsin B.^[^ ^26^ ^]^ Whereas it was found that the conjugated PROTACs molecules were difficultly liberated to initial formation totally, and these processes were also time‐consuming and compromised their enzyme‐like activity,^[^ ^25^ ^]^ partly accounting for their auxiliary roles in tumor immunometabolic intervention and photodynamic therapy. What is more, the use of nanoparticle‐assisted PROTACs in directly dismantling oncogenic proteins for tumor treatment remains largely unexplored.^[^ ^26^, ^27^ ^]^ Apart from playing the role of transport vehicle, the intrinsic property of specific nanocarriers might also contribute to potentiating the PROTACs' function through additive mechanisms in mediating the microenvironment of the disease sites. However, to the best of our knowledge, currently, there is no report involved in the potential interaction between carriers and PROTACs molecules in this regard (i.e., the nanocarriers themselves also mediate the microenvironment of the disease sites to further amply effects of loaded PROTACs via specific bio‐mechanisms).

**Scheme 1:**
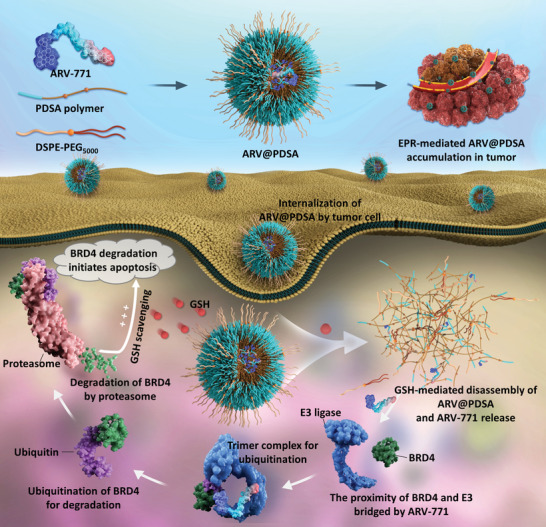
Schematic illustration of the construction of ARV@PDSA and the Nano‐PROTACs strategy for enhanced targeted protein degradation. PROTACs are engineered by encapsulation in the redox‐responsive PDSA nanoparticles whose surface is modified with DSPE‐PEG_5000_ for prolonged blood circulation and efficient tumor accumulation via the enhanced permeability and retention effect. The disassembly of ARV@PDSA activated by a high concentration of intracellular glutathione facilitates the release of the encapsulated PROTACs in cancer cells. Subsequently, the released PROTACs effectively hijack the ubiquitin‐proteasome system to degrade the intracellular BRD4, which leads to enhanced anti‐tumor efficacy.

Herein, we provide proof‐of‐principle design and use of nanoengineered PROTACs (Nano‐PROTACs) to augment the targeted degradation of proteins associated with tumor progression via a specific glutathione (GSH)‐scavenging strategy (**Scheme** **1**). We constructed the ARV@PDSA Nano‐PROTACs from redox‐responsive poly(disulfide amide) (PDSA) polymer to encapsulate the BRD4 degrader ARV‐771 PROTACs and from lipid‐polyethylene glycol (lipid‐PEG) to improve biocompatibility. Without the need for additional chemical conjugation, such a straightforward yet innovative strategy for the construction of redox‐responsive ARV@PDSA Nano‐PROTACs not only substantially enhances accumulation/responsive‐release in tumor regions and bioavailability but also concomitantly scavenges GSH to neutralize the microenvironment and amplify the anti‐tumor efficacy of ARV‐771 via c‐Myc‐related ferroptosis and cell cycle arrest pathways,^[^ ^28^, ^29^ ^]^ potentiating future clinical translation.

## 2 Results

### 2.1 Synthesis and Characterization of Redox‐Responsive ARV@PDSA Nano‐PROTACs

The ARV@PDSA Nano‐PROTACs were designed on the basis of the following considerations: i) efficient encapsulation in PDSA nanoparticles to enhance accumulation in targeted cells; ii) redox‐responsive release to improve bioavailability in tumor cells while minimizing off‐target side effects; iii) increased blood circulation time to facilitate the tumor accumulation of ARV@PDSA via the enhanced permeability and retention effect; iv) concomitantly scavenge GSH and in turn reinforce the BRD4 degradation for improved tumor therapy.

**Figure 1:**
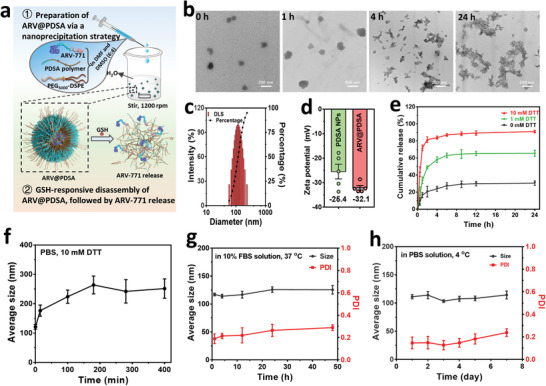
Synthesis and characterization of ARV@PDSA. a) Schematic illustration of the synthesis of ARV@PDSA and GSH‐responsive release of ARV‐771 from ARV@PDSA. b) TEM images of ARV@PDSA after incubation with 10 mm DTT for 0, 1, 4, and 24 h. Scale bar, 200 nm. c) DLS size of ARV@PDSA. d) Zeta potentials of PDSA nanoparticles and ARV@PDSA. e) Release profiles of ARV‐771 from ARV@PDSA incubated with different DTT concentrations. f) Time‐dependent DLS size of ARV@PDSA in PBS containing 10 mm DTT. g) Time‐dependent DLS size and polydispersity index (PDI) of ARV@PDSA in PBS containing 10% FBS at 37 °C and h) PBS at 4 °C. Data are presented as mean ± SD.

The chemical structure of redox‐responsive PDSA polymer synthesized via a one‐step polycondensation reaction of l‐cystine dimethyl ester dihydrochloride and adipoyl chloride was validated by proton nuclear magnetic resonance (Figure S1a,b, Supporting Information).^[^ ^30^ ^]^ Subsequently, PDSA polymeric nanoparticles were obtained through an optimized self‐assembly process of PDSA polymer in an aqueous solution, during which the hydrophobic ARV‐771 PROTACs were readily encapsulated into the core of PDSA nanoparticles (**Figure** **1**a). To enhance colloidal stability and prolong blood circulation, the surface of PDSA nanoparticles was coated with amphipathic 1,2‐distearoyl‐*sn*‐glycero‐3‐phosphoethanolamine‐*N*‐[methoxy(polyethylene glycol)] (DSPE‐PEG);^[^ ^30^, ^31^ ^]^ the resulting PEGylated PDSA nanoparticles with the encapsulated ARV‐771 were designated ARV@PDSA. Dynamic light scattering (DLS) analysis showed that the size of ARV@PDSA in phosphate‐buffered saline (PBS) solution was 118 nm, which was similar to that of ARV@PDSA observed by transmission electron microscopy (TEM) (Figure 1b,c). High‐performance liquid chromatography (HPLC) showed that the loading capacity [defined as (mass of loaded ARV‐771/ mass of PDSA) × 100%)] and loading efficiency [defined as (mass of loaded ARV‐771/mass of total ARV‐771) × 100%) of ARV‐771 at a feeding ratio of 1:12.5 (ARV‐771:PDSA)] were 6.8% and 85.3%, respectively. In addition, the loading of ARV‐771 in PDSA nanoparticles led to a slight change in zeta potential (Figure 1d).

Next, we studied the redox‐responsive release of ARV‐771 from ARV@PDSA Nano‐PROTACs. To simulate the redox environment of cancer cells, we used PBS solutions containing different concentrations of dithiothreitol (DTT) reducing agent.^[^ ^30^, ^31^ ^]^ The release of ARV‐771 in PBS leveled off 12 h after the release starting point, at which time the release efficiency was only 25.5%. By contrast, the release efficiencies of ARV‐771 in PBS containing 1 or 10 mm DTT at 12 h were 65.8% and 88.7%, respectively (Figure 1e), much higher than that without the DTT treatment. These results demonstrate that PDSA stabilizes the encapsulated ARV‐771 and selectively releases ARV‐771 in a redox‐responsive manner, supporting the promise of the Nano‐PROTACs strategy for transporting PROTACs into targeted cancer cells and enabling the enhanced release of PROTACs inside the cancer cells.

To further confirm the redox‐responsive release of ARV‐771 from Nano‐PROTACs, we investigated the changes in size and morphology of ARV@PDSA under the same conditions used in the ARV‐771 release study. DLS analysis showed that the size of ARV@PDSA in PBS containing 10 mm DTT rapidly increased from 110 to 180 nm within the first 30 min and continuously increased to ≈260 nm at 3 h (Figure 1f), suggesting the disintegration of ARV@PDSA. Moreover, TEM images showed that the spherical ARV@PDSA gradually disintegrated and finally became irregular debris after 24 h incubation in PBS with 10 mm DTT (Figure 1b). In contrast, ARV@PDSA showed excellent colloidal stability in both physiological and storage environments as determined by the DLS size in PBS containing 10% fetal bovine serum (FBS) at 37 °C over a period of 48 h (Figure 1g) or in PBS at 4 °C over a period of 6 days (Figure 1h). Together, these results demonstrate that the redox‐responsive degradation of PDSA nanoparticles selectively releases encapsulated PROTACs.

### 2.2 ARV@PDSA Nano‐PROTACs Promote the Degradation of BRD4 In Vitro

**Figure 2:**
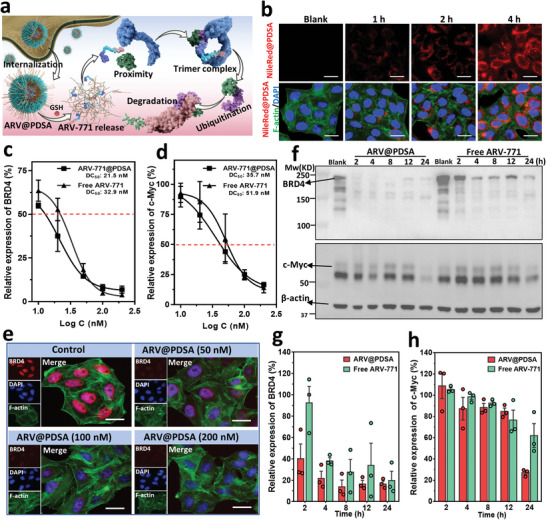
Targeted degradation of BRD4 and downregulation of c‐Myc by ARV@PDSA. a) Schematic illustration of internalization of ARV@PDSA, GSH‐responsive release of ARV‐771 from ARV@PDSA, and BRD4 degradation by hijacking the ubiquitin‐proteasome system. b) Representative fluorescence microscope images of uptake of NileRed‐labeled PDSA nanoparticles by HeLa cells after incubation for different periods of time. Scale bars, 25 µm. Concentration‐dependent degradation of c) BRD4 and d) c‐Myc in HeLa cells. The half maximal degradation concentration (DC50) of ARV@PDSA and free ARV‐771 were derived by fitting the levels of BRD4 and c‐Myc expression versus ARV‐771 concentration using a sigmoidal model. Data are shown as mean ± s.e.m. (*n* = 3). e) Representative immunofluorescence images of BRD4 protein in HeLa cells after incubation with different concentrations of ARV@PDSA for 24 h. Scale bars, 25 µm. f) Western blot analysis of the expression levels of BRD4 and c‐Myc in HeLa cells after incubation with ARV@PDSA and free ARV‐771 at the ARV‐771 concentration of 100 nm for different periods of time. Quantification of band intensity of g) BRD4 and h) c‐Myc from (f). Data are shown as mean ± s.e.m. (*n* = 3).

We subsequently explored the potential of ARV@PDSA Nano‐PROTACs for the selective degradation of targeted proteins in vitro (**Figure** **2**a). To investigate the cellular uptake of ARV@PDSA and achieving proteolysis of BRD4,^[^ ^22^ ^]^ we first labeled PDSA nanoparticles with Nile Red and tested them in human cervical cancer cells (HeLa) and murine melanoma cells (B16F10). Fluorescence microscopy images showed the red fluorescence signals distributed over the cell cytoplasm and the uptake of ARV@PDSA Nano‐PROTACs was in a time‐dependent manner (Figure 2b and Figures S2 and S3, Supporting Information). Of note, the cellular uptake of ARV@PDSA could be observed after only 1 h incubation, suggesting that the Nano‐PROTACs strategy efficiently and quickly transports ARV‐771 PROTACs into targeted cancer cells.

We next studied the ARV@PDSA Nano‐PROTACs‐mediated degradation of intracellular BRD4 and its downstream effector, the oncogene c‐Myc.^[^ ^10^, ^32^ ^]^ Our data showed that although both ARV@PDSA and free ARV‐771 treatments resulted in dose‐dependent degradation of BRD4 in HeLa cells (Figure 2c), the half maximal BRD4 degradation concentration (DC50), derived by fitting the level of BRD4 degradation versus ARV‐771 concentration using a sigmoidal model, of ARV@PDSA was 21.5 nm (Figure 2c), lower than that of free ARV‐771 (32.9 nm). Notably, ARV@PDSA enhanced BRD4 degradation when compared to the free ARV‐771, especially at low concentrations (10 and 20 nm). The enhanced BRD4 degradation mainly resulted from the efficient intracellular accumulation of ARV‐771 PROTACs assisted by the Nano‐PROTACs strategy. Immunofluorescence (IF) staining confirmed that ARV@PDSA led to a dose‐dependent degradation of BRD4 and increasing the concentrations gradually decreased intranuclear BRD4 signal intensity (Figure 2e and Figure S4, Supporting Information), which was also observed in B16F10 cells (Figure S5, Supporting Information). Moreover, the BRD4 degradation induced by ARV@PDSA or ARV‐771 downregulated the expression of c‐Myc, whose expression was also reflected in a dose‐dependent manner (Figure 2d). Remarkably, the DC50 of ARV@PDSA for c‐Myc was fitted to be 35.7 nm, which was lower than that of free ARV‐771 (51.9 nm, Figure 2d). Together, these in vitro results demonstrate that ARV@PDSA treatment not only facilitated the BRD4 degradation but also efficiently drugged the undruggable oncogene c‐Myc, again highlighting the promise of the Nano‐PROTACs strategy for anti‐tumor therapy.

Next, we studied the kinetics of BRD4 degradation. Strikingly, ARV@PDSA degraded ≈60% of intracellular BRD4 in the first 2 h, leveling off 8 h post‐treatment at ≈80% in HeLa cells (Figure 2f,g). In comparison, free ARV‐771 led to only ≈10% BRD4 degradation in the first 2 h. Of note, though BRD4 degraded mainly in the first 2–4 h, the downregulation of c‐Myc primarily occurred between 12 and 24 h post‐ARV@PDSA treatment, later than BRD4 degradation (Figure 2f,h). Those findings could be explained by the delayed responses in the intracellular signal transduction between BRD4 and c‐Myc proteins. Similar results were found in B16F10 murine melanoma cells, a second type of in vitro cell model (Figure S6, Supporting Information). Meanwhile, our data showed that empty PDSA nanoparticles had no influence on the expression of BRD4 and c‐Myc (Figure S7, Supporting Information), indicating that their degradation in HeLa and B16F10 cells primarily resulted from the released ARV‐771 PROTACs. Taken together, these results showed that the Nano‐PROTACs strategy enhances the efficiency of BRD4 degradation by improving the permeability and accumulation of ARV‐771 PROTACs in cancer cells.

### 2.3 GSH‐Scavenging ARV@PDSA Nano‐PROTACs Enhanced Anti‐Cancer Efficacy In Vitro

**Figure 3:**
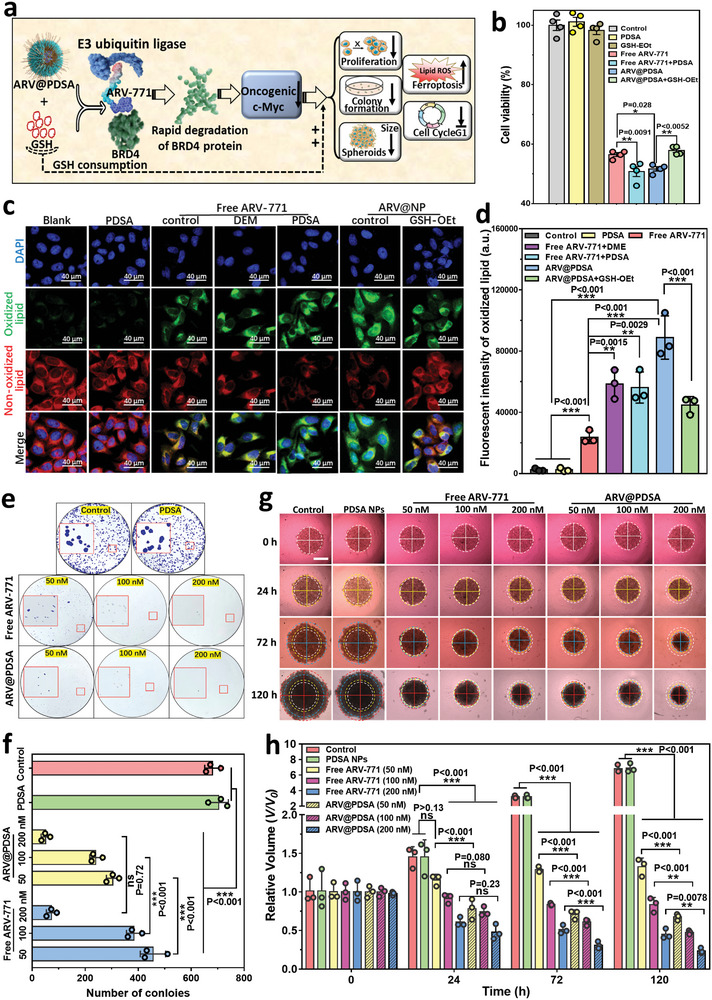
In vitro anti‐cancer effect of ARV@PDSA. a) Schematic illustration of enhanced in vitro anti‐tumor effect of ARV‐771 by PDSA nanoparticles. b) Cell viability of HeLa cells after treatment with ARV@PDSA or free ARV‐771 for 24 h in different conditions. Data are shown as mean ± s.e.m. (*n* = 4), and analyzed by one‐way ANOVA with a Tukey post hoc test. c) Representative fluorescence microscope images of oxidized and non‐oxidized lipids in HeLa cells after the treatment with ARV@PDSA or free ARV‐771 for 24 h at an ARV‐771 concentration of 1000 nm in different conditions. d) Quantification of fluorescence intensity of oxidized lipid in HeLa cells from (c). Data are shown as mean ± SD (*n* = 3), and analyzed by one‐way ANOVA with a Tukey post hoc test. e) Representative microscope images of colony formation of HeLa cells stained with crystal violet after treatment with various concentrations of ARV@PDSA and free ARV‐771. f) Quantification of colonies in the various groups from (e). Data are shown as mean ± SD (*n* = 3), and analyzed by two‐way ANOVA with Sidak's test. g) Representative microscope images of HeLa tumor spheroids after treatment with various concentrations of ARV@PDSA and free ARV‐771. Scale bar, 500 µm. h) Quantification of the relative volume of tumor spheroids from (g). Data are shown as mean ± SD (*n* = 3), and analyzed by two‐way ANOVA with Sidak's test. *p* < 0.05 is considered significantly different, the significance levels are*p < 0.05, **p < 0.01, ***p < 0.001, and ns denotes not significant.

To evaluate the anti‐cancer efficacy of ARV@PDSA in vitro, we utilized 2D cell culture and 3D multicellular tumor spheroid models of HeLa cervical cancer cells (**Figure** **3**a). The MTT assay showed no cytotoxicity of blank PDSA nanoparticles in HeLa cells, rendering such nanoparticles biocompatible platforms for ARV‐771 PROTACs delivery (Figure S8, Supporting Information). In contrast, ARV@PDSA showed an obvious concentration‐dependent cell‐killing effect on HeLa cells (Figure S9, Supporting Information), especially at a concentration of 1000 nm of ARV‐771 (Figure 3b). More importantly, the cytotoxicity of ARV@PDSA was higher than that of free ARV‐771, demonstrating that the Nano‐PROTACs strategy enhances the anti‐tumor efficacy of ARV‐771 PROTACs by improving their permeability and exposure to cancer cells. This enhanced cytotoxicity was also observed in B16F10 cells (Figure S10, Supporting Information). We found that while HeLa cells have a slightly lower expression of BRD4 than B16F10 cells, they have a much higher expression of c‐Myc (Figure S11a,b, Supporting Information). Furthermore, treatment with ARV‐771 led to a greater reduction in BRD4 and c‐Myc expression in HeLa cells than in B16F10 cells (Figure S11c, Supporting Information), indicating that BRD4 proteolysis in HeLa cells is more sensitive to ARV‐771 treatment. This effect led to a stronger anticancer effect of ARV@PDSA on HeLa cells compared to B16F10 cells. Moreover, these results also suggest that treatment with ARV@PDSA showed the enhanced downregulation of the c‐Myc protein, which in turn led to a greater cell‐killing effect.^[^ ^10^ ^]^

In addition to acting on the BRD4‐c‐Myc axis for enhanced cell apoptosis, BRD4 degradation also induces both ferroptosis^[^ ^33^ ^]^ and cell‐cycle arrest.^[^ ^34^ ^]^ ARV@PDSA treatment of HeLa cells led to higher levels of lipid peroxidation than free ARV‐771 treatment (Figure 3c,d), suggesting ARV@PDSA induced more robust cell ferroptosis. In addition, cell cycle analysis showed that ARV@PDSA treatment induced a significant reduction in the number of cells in the S phase, which was associated with G1 arrest (Figure S12, Supporting Information), suggesting an in vitro anti‐proliferation effect. The enhanced cell ferroptosis and cell cycle arrest might be resulted from the persistent consumption of intracellular GSH by the abundant disulfide bonds in the matrix of PDSA nanoparticles.^[^ ^29^ ^]^ We further used diethyl maleate (DEM) or blank PDSA to exclusively exhaust intracellular GSH similar to ARV@PDSA, and glutathione‐reduced ethyl ester (GSH‐OEt) to replenish the intracellular GSH consumed by PDSA in the investigation of cell viability and ferroptosis (Figure 3b,c). Remarkably, the preincubation of PDSA could obviously reinforce the cell‐killing effect of free ARV‐771 to HeLa, and the refill of the intracellular GSH decreased the anti‐cancer effect of ARV@PDSA (Figure 3b). The intracellular GSH consumption by PDSA was also performed in lipid peroxidation in ARV‐771‐induced ferroptosis (Figure 3c,d).

We next investigated the anti‐proliferation effect of ARV@PDSA on HeLa cells using a colony formation assay.^[^ ^35^ ^]^ Consistent with the findings of the cell viability, no significant difference was found in the numbers or sizes of HeLa colonies between PDSA nanoparticles and control groups (Figure 3e,f). In contrast, both ARV@PDSA and free ARV‐771 displayed concentration‐dependent inhibition effects on the size and number of HeLa colonies. Remarkably, ARV@PDSA treatment produced greater inhibition than free ARV‐771 at concentrations of 50 and 100 nm. The same results were observed in B16F10 cells (Figure S13, Supporting Information). Similar to the cytotoxicity assay, this inhibition effect on HeLa cells was greater than on B16F10 cells (Figure 3b and Figure S10, Supporting Information).

We further studied the anti‐tumor effect of ARV@PDSA by using a 3D multicellular tumor spheroid model of HeLa cervical cancer, which more closely mimics the in vivo tumor microenvironment in terms of cellular morphology and intercellular matrix interaction.^[^ ^36^ ^]^ Consistent with the results of cytotoxicity and colony assays, control PDSA showed negligible effects on the size and volume of tumor spheroids during treatment (120 h) (Figure 3g,h). Impressively, although the treatment of tumor spheroids with free ARV‐771 and ARV@PDSA at all concentrations showed an obvious anti‐tumor effect compared with the control, ARV@PDSA treatment induced a more remarkable anti‐tumor effect when compared with their initial volume. The superior anti‐tumor efficacy of ARV@PDSA in the 3D tumor spheroid model might depend on its exceptional ability to penetrate the cell membrane and the intercellular space of tumor spheroids. The ARV@PDSA Nano‐PROTACs not only effectively infiltrate the intercellular space to reach deep tumor regions but also efficiently penetrate the cell membrane to increase the intracellular ARV‐771 accumulation. In contrast, the lipophilic nature of ARV‐771 allows the molecules to diffuse only to the superficial area of tumor spheroids and inhibits drug internalization through the cell membrane, hindering effective accumulation in HeLa cells.

On the basis of these in vitro findings, we conclude that ARV@PDSA Nano‐PROTACs, with their desirable size,^[^ ^37^, ^38^ ^]^ favorable colloidal stability, unique GSH‐scavenging ability, and cell membrane permeability, enhance the capability of ARV‐771 to reach the deep tumor spheroid region and substantially enhance tumor killing.^[^ ^23^ ^]^

### 2.4 In Vivo Biodistribution in Tumor‐Bearing Mice

**Figure 4:**
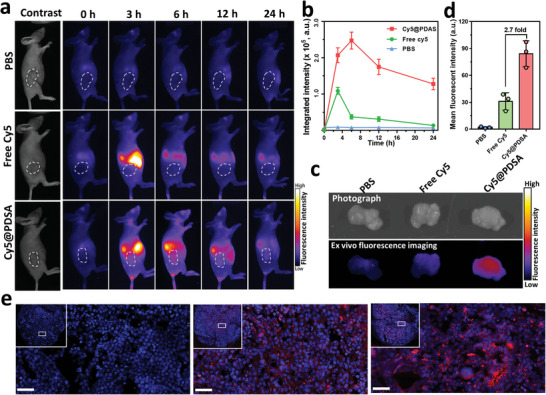
In vivo biodistribution of PDSA nanoparticles. a) Time‐dependent near‐infrared fluorescence images of Cy5@PDSA and free Cy5 in HeLa tumor‐bearing athymic nude mice. The tumor regions are identified by the white dotted circles. b) Quantification of fluorescence intensity in tumor regions from (a). c) Ex vivo near‐infrared fluorescence imaging of intra‐tumoral Cy5@PDSA and free Cy5 24 h after intravenous administration. d) Quantification of fluorescence intensity in tumors from (c). e) Fluorescence microscope images of tumor tissue sections after Cy5@PDSA and free Cy5 treatments. Inset shows a large area of the tumor tissue section. Scale bars, 50 µm. Data are shown as mean ± SD (*n* = 3).

The in vitro results led us to study the in vivo biodistribution of these PDSA‐based nanoparticle delivery platforms. We used Cy5‐labeled PDSA nanoparticles (Cy5@PDSA) to treat HeLa tumor‐bearing athymic nude mice. Near‐infrared fluorescence imaging showed that the fluorescence intensity of the tumor gradually increased within the first 6 h after systemic administration of Cy5@PDSA (**Figure** **4**a,b),^[^ ^39^ ^]^ indicating that Cy5@PDSA efficiently accumulated in the tumor. Mice were sacrificed 24 h post‐administration, and the tumors were harvested for ex vivo fluorescence imaging. The Cy5 fluorescence intensity of the tumor treated by Cy5@PDSA was 2.7 times higher than that produced by free Cy5 treatment. (Figure 4c,d). The effective accumulation of Cy5@PDSA in the tumor region was also demonstrated by fluorescence microscopy of tumor tissue sections (Figure 4e). Collectively, these results demonstrate that Cy5@PDSA efficiently accumulates in the tumor region after systemic administration.

### 2.5 In Vivo Anti‐Tumor Efficacy of ARV@PDSA Nano‐PROTACs

**Figure 5:**
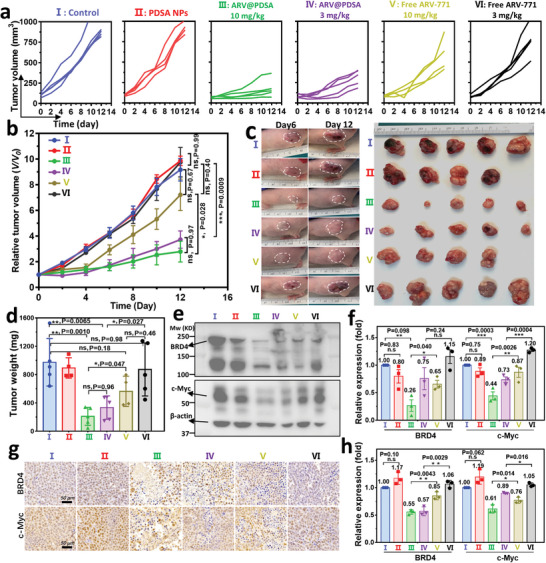
In vivo anti‐tumor efficacy of ARV@PDSA in HeLa tumor‐bearing athymic nude mice. a) Individual and b) average tumor growth curves of various treatment groups. Data are shown as mean ± s.e.m. (*n* = 4 or 5), and analyzed by one‐way ANOVA with a Tukey post hoc test. Data are shown as mean ± s.e.m. (*n* = 4 or 5). c) Representative photographs of tumor‐bearing mice 6 and 12 days after various treatments (left panel). The tumor regions are indicated by the white dotted circles. The tumors were harvested 12 days after various treatments (right panel). d) Tumor weight of different groups after 12‐day treatment. Data are shown as mean ± s.e.m. (*n* = 4 or 5), and analyzed by one‐way ANOVA with a Tukey post hoc test. e) Representative western blot assay of BRD4 and c‐Myc expression in tumor tissues at the end of each treatment. f) Quantification of the band intensity of BRD4 and c‐Myc from (e). Data are shown as mean ± s.e.m. (*n* = 3), and analyzed by one‐way ANOVA with a Tukey post hoc test. g) Representative immunohistochemistry staining assay of BRD4 and c‐Myc in tumor tissue sections after various treatments. h) Quantification of BRD4 and c‐Myc intensities from (g). Data are shown as mean ± s.e.m. (*n* = 3), and analyzed by one‐way ANOVA with a Tukey post hoc test. *p* < 0.05 is considered significantly different, the significance levels are *p < 0.05, **p < 0.01, ***p < 0.001, and ns denotes not significant. (I: Control, II: PDSA, III: ARV@PDSA (10 mg kg^−1^), IV: ARV@PDSA (3 mg kg^−1^), V: Free ARV‐771 (10 mg kg^−1^), VI: Free ARV‐771 (3 mg kg^−1^)).

All the above results encouraged us to assess the anti‐tumor efficacy of the ARV@PDSA Nano‐PROTACs strategy in HeLa and B16F10 subcutaneous xenograft mouse models. HeLa tumor‐bearing nude mice were randomly divided into six groups: I) control; II) PDSA; III) ARV@PDSA (10 mg ARV‐771/kg); IV) ARV@PDSA (3 mg ARV‐771/kg); V) free ARV‐771 (10 mg ARV‐771/kg); and VI) free ARV‐771 (3 mg/kg), in which two different doses of ARV@PDSA and free ARV‐771 were administered to compare their dose‐dependent therapeutic efficacy. After intravenous injection, we measured the tumor volume and body weight of mice every other day over 12 days (**Figure** **5**a–c). One day after the last treatment, tumors were harvested to determine the tumor inhibition rates (TIR). Treatment of mice with PDSA nanoparticles showed no therapeutic advantage over the control (Figure 5a–d). Moreover, a moderate anti‐tumor efficacy of free ARV‐771 treatment was observed, as the TIRs were 42.1% and 10.3% for 10 (V) and 3 mg kg^−1^ (VI) groups, respectively (Figure S14a, Supporting Information). In contrast, ARV@PDSA showed superior anti‐tumor efficiency with TIRs of 78.4% and 65.6% of the 10 (III) and 3 mg kg^−1^ (IV) groups, respectively (Figure S14a, Supporting Information). The superior therapeutic efficacy of ARV@PDSA was mainly caused by a combination of improved pharmacokinetics, effective tumor accumulation, and GSH‐scavenging strategy‐amplified antitumor effects. Specifically, after blood circulation, ARV@PDSA accumulating in the tumor region could be readily taken up by HeLa tumor cells, followed by the redox‐responsive release of ARV‐771 from ARV@PDSA in the cells and GSH‐scavenging within these tumor cells.

To further investigate the anti‐tumor mechanism of ARV@PDSA, we determined the expressions of BRD4 and c‐Myc in tumor samples harvested one day after the last treatment. Our in vivo data showed that free ARV‐771 elicited only moderate BRD4 degradation (35%) and a slight c‐Myc reduction (13%) at the dose of 10 mg kg^−1^, and showed negligible effects on these two proteins at 3 mg kg^−1^ (Figure 5e,f). By contrast, treatment with 10 mg kg^−1^ ARV@PDSA showed significant degradation of BRD4 (74%) and obvious downregulation of c‐Myc (56%), notably more than free ARV‐771 treatment. In addition, these two proteins underwent dose‐dependent degradation after ARV@PDSA treatment; treatment with a lower dose of ARV@PDSA (3 mg kg^−1^) induced BRD4 degradation (25%) and c‐Myc downregulation (27%) (Figure 5f). Immunohistochemistry (IHC) staining assay showed similar results that the highest BRD4 and c‐Myc reduction of tumor tissue were observed after treatment with 10 mg kg^−1^ ARV‐771@PDSA (Figure 5g,h). Collectively, these results demonstrate that treatment with ARV@PDSA not only substantially inhibited tumor growth by inhibiting BRD4 and c‐Myc but also showed a superior anti‐tumor therapy strategy than free PROTACs. It is worth mentioning that the body weight of mice showed no significant variations between groups during treatment, suggesting no systemic toxicity of ARV@PDSA (Figure S15a, Supporting Information). In addition, the hematoxylin and eosin (H&E) staining of tissue sections of the main organs, including the heart, liver, spleen, lung, and kidney, showed no apparent damage (Figure S16, Supporting Information), suggesting biocompatibility.

**Figure 6:**
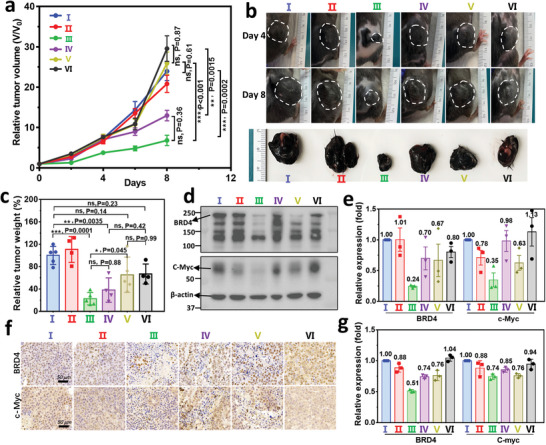
In vivo anti‐tumor efficacy of ARV@PDSA in B16F10 tumor‐bearing C57BL/6 mice. a) Average tumor growth curves of various treatment groups. Data are shown as mean ± s.e.m. (*n* = 4 or 5), and analyzed by one‐way ANOVA with a Tukey post hoc test. b) Representative photographs of tumor‐bearing mice 4 and 8 days after various treatments (up panel). The tumor regions are indicated by the white dotted circles. The tumors were harvested 8 days after various treatments (down panel). c) Tumor weight of different groups relative to the control after 8‐day treatment. Data are shown as mean ± s.e.m. (*n* = 4 or 5), and analyzed by one‐way ANOVA with a Tukey post hoc test. d) Representative western blot assay of BRD4 and c‐Myc expression in tumor tissues at the end of each treatment. Data are shown as mean ± s.e.m. (*n* = 3). e) Quantification of the band intensity of BRD4 and c‐Myc from (d). Data are shown as mean ± s.e.m. (*n* = 3). f) Representative immunohistochemistry staining assay of BRD4 and c‐Myc in tumor tissue sections after various treatments. g) Quantification of BRD4 and c‐Myc intensities from (f). Data are shown as mean ± s.e.m. (*n* = 3). *p* < 0.05 is considered significantly different, the significance levels are *p < 0.05, **p < 0.01, ***p < 0.001, and ns denotes not significant. (I: Control, II: PDSA, III: ARV@PDSA (10 mg kg^−1^), IV: ARV@PDSA (3 mg kg^−1^), V: Free ARV‐771 (10 mg kg^−1^), VI: Free ARV‐771 (3 mg kg^−1^)).

In addition to ovarian cancer, recent studies showed that the increased expression of BRD4 was strongly associated with the initiation and progression of melanoma through promoting the expression of oncogenes, such as c‐Myc, as transcriptional regulators or epigenetic readers.^[^ ^4^, ^6^ ^]^ Those findings suggest that targeted degradation of BRD4 could be a promising strategy for melanoma treatment.^[^ ^40^ ^]^ To this end, we investigated the anti‐tumor efficacy of ARV@PDSA in B16F10 melanoma‐bearing C57BL/6 mice. Mice were divided into six groups (the same as those used for HeLa tumor treatment; **Figure** **6**a,b). Consistent with the results of Nano‐PROTAC‐mediated treatment of HeLa tumor mice, moderate anti‐tumor efficacy of free ARV‐771 treatment was observed, as the TIRs were 34.6% and 33.1% for 10 (V) and 3 mg kg^−1^ (VI) groups, respectively (Figure 6c and Figure S14b, Supporting Information). In contrast, treatment with ARV@PDSA significantly inhibited tumor growth, as those TIRs were 77.4% and 61.8% for 10 (III) and 3 mg kg^−1^ (IV) groups, respectively (Figure S14b, Supporting Information). ARV@PDSA‐mediated BRD4 degradation and c‐Myc downregulation of B16F10 tumor cells were also demonstrated by western blot (Figure 6d,e) and IHC staining (Figure 6f,g) assays. Similarly, treatment of ARV@PDSA showed no systemic toxicity as observed by the mouse body weight (Figure S15b, Supporting Information) and H&E staining (Figure S17, Supporting Information) of the main organs. Collectively, these results demonstrate that ARV@PDSA shows superior anti‐tumor efficacy by efficiently inhibiting intracellular BRD4 and c‐Myc expression in both HeLa and B16F10 tumor‐bearing mouse models, highlighting the significance of our Nano‐PROTACs strategy to expand the therapeutic landscape for the treatment of other diseases.

## 3 Discussion

PROTACs are an emerging class of promising therapeutic modalities that selectively degrade intracellular proteins of interest by hijacking the UPS. However, the lack of techniques to efficiently transport these degraders to targeted cells and consequently the potential toxicity of PROTACs limit their clinical applications. In the current study, we designed a strategy based on nanoengineered PROTACs (Nano‐PROTACs) that enhances the targeted degradation of proteins of interest and the druggability of oncogenic proteins for cancer therapy. Specifically, we nanoengineered ARV‐771 by encapsulating these BRD4‐degrading PROTACs into PEGylated PDSA nanoparticles with a high PROTACs loading capacity. Such a straightforward process for the preparation of ARV@PDSA Nano‐PROTACs avoids complicated synthetic routes, highlighting its promise for future clinical translation. Additionally, the Nano‐PROTACs strategy might address the challenges associated with the clinical use of free ARV‐771 PROTACs including poor water solubility, limited cell membrane permeability, high clearance rate, short half‐life, and limited anti‐tumor efficacy in vivo. Remarkably, the ARV@PDSA Nano‐PROTACs strategy not only improves the pharmacokinetic profile of free ARV‐771 but also leverages the intrinsic GSH scavenging capacity of PDSA carriers, achieving superior anti‐tumor efficacy compared to free ARV‐771 treatment. The key findings regarding our ARV@PDSA Nano‐PROTACs strategy are: i) efficient release of encapsulated ARV‐771 PROTACs in a redox‐responsive manner and facilitated accumulation and bioavailability in tumor cells, ii) effective induction of degradation of the tumor‐driver BRD4 and, in turn, downregulation of the expression of the intracellular c‐Myc oncogenic protein, iii) enhanced anti‐tumor efficacy by increasing cytotoxicity, inhibiting tumor colony formation and the volume of tumor spheroids in comparison to those of free ARV‐771 treatment, and iv) significant inhibition of tumor growth and reduction of tumor volume in both HeLa and B16F10 tumor‐bearing mouse models by substantially reducing the BRD4 and c‐Myc expression in tumor cells.

Biomedical applications of nanoengineered PROTACs are still in their infancy. To date, PROTACs‐loaded nanoparticles have mostly been studied preliminary in vitro in several studies. For example, Saraswat et al. used PLGA polymeric nanoparticles to improve the solubility and cell membrane permeability of ARV‐825 PROTACs, and thereby, enhanced the targeted protein degradation in pancreatic cancer cells.^[^ ^23^ ^]^ Additionally, Chen et al. developed a lipid nanoparticle‐based system to deliver pre‐fused PROTACs to improve the degradation of heat shock protein in androgen‐sensitive human prostate adenocarcinoma cells.^[^ ^22^ ^]^ Despite these achievements, key challenges, such as targeted protein degradation and robust therapeutic efficacy, in in vivo models remain elusive. A recent study by Zhang et al. addressed these problems using a semiconducting polymeric nanoparticle‐mediated PROTACs strategy in conjunction with photodynamic therapy for in vivo cancer immunometabolic therapy.^[^ ^26^ ^]^ Although the treatment with polymeric nanoparticle‐mediated PROTACs selectively degraded the immunometabolism‐associated proteins in vivo, the anti‐tumor efficacy of such a treatment remained unsatisfactory. Our approach instead employed the unique redox‐responsive Nano‐PROTACs, which can directly target the essential yet intractable BRD4‐c‐Myc axis. Its universal therapeutic potential was demonstrated by remarkable in vivo anti‐tumor efficacy against both HeLa and B16F10 tumors associated with c‐Myc‐derived malignancies.^[^ ^10^ ^]^

PROTACs in their free drug form must be administered at a high dose to achieve desirable therapeutic efficacy;^[^ ^1^, ^3^ ^]^ the dose may reach 100 mg kg^−1^, which is tenfold to 20‐fold higher than commonly used chemotherapeutics such as doxorubicin. Such a high dose of PROTACs may lead to systemic side effects and undermine the promise of a PROTACs‐mediated therapeutic strategy. In contrast, in the current study, we observed remarkable anti‐tumor efficacy of BRD4‐degrading ARV@PDSA Nano‐PROTACs, even when lowering the dose range to 3 and 10 mg ARV‐771/kg, within the tolerated range of commonly studied chemotherapeutics.^[^ ^41^ ^]^ Indeed, biosafety analysis in the current study showed no significant variations in mouse body weight, and no obvious difference in H&E staining of main organs between groups, further confirming the biocompatibility of the ARV@PDSA Nano‐PROTACs for tumor therapy. In terms of the nanoparticle platform, PDSA can be synthesized via a one‐step polycondensation reaction of l‐cystine dimethyl ester dihydrochloride and adipoyl chloride, which enables large‐scale synthesis. Additionally, the abundant disulfide bonds in the PDSA structure react with intracellular GSH, releasing the encapsulated ARV‐771 in cancer cells. In conjunction with the released ARV‐771, the consumption of GSH may not only strengthen the antiproliferative effect of BRD4 proteolysis but also enhance the cytotoxicity triggered by c‐Myc downregulation through a canonical redox‐mediated pathway,^[^ ^29^, ^42^ ^]^ highlighting the potential of PDSA nanoparticles in Nano‐PROTACs for cancer therapy. In addition, nanoparticulate delivery platforms conjugating with suitable targeting ligands can be used to improve the accumulation and specificity of PROTACs in the targeted cancer cells. Furthermore, the targeted delivery of dual therapeutics, such as PROTACs and chemotherapeutic drugs, or PROTACs and immunotherapeutic drugs, that synergistically act on different anti‐tumor pathways may also increase the efficacy of the nanotherapeutics. However, to realize the clinical translation of the Nano‐PROTACs therapeutic strategy, several essential considerations remain:^[^ ^16^, ^43^, ^44^ ^]^ i) optimization of the size, charge, and surface targeting ligands of Nano‐PROTACs for the disease being targeted, ii) detailed evaluation of the therapeutic efficacy of Nano‐PROTACs compared with free PROTACs alone, and iii) systemic assessment of toxicity, biosafety, immunogenicity, and clearance of the Nano‐PROTACs in large animal models.

## 4 Conclusion

In summary, this study presents a GSH‐scavenging Nano‐PROTACs strategy that not only improves the bioavailability and accumulation of PROTACs in diseased cells but also enhances targeted protein degradation and improves druggability to target undruggable oncogenic proteins for tumor treatment. The GSH‐scavenging Nano‐PROTACs technology holds exciting promise for the treatment of a broad range of diseases by attacking their pathogenic proteins. This study may also pave the way for more smart PROTAC‐nanotechnology strategies that intelligently transfer the intrinsic properties of nanocarriers to amplify the therapeutic effects of loaded PROTACs, greatly inspiring future studies.

## 5 Experimental Section

### Materials

All the chemicals and reagents used for the synthesis of PDSA, including l‐Cystine dimethyl ester dihydrochloride ((H‐Cys‐OMe)2.2HCl), adipoyl chloride, triethylamine, DEM, glutathione reduced ethyl ester (GSH‐OEt), dimethyl sulfoxide (DMSO), dimethylformamide (DMF), and ethyl ether, were purchased from Sigma‐Aldrich without further purification unless otherwise stated. 1,2‐Distearoyl‐*sn*‐glycero‐3‐phosphoethanolamine‐*N*‐[methoxy(polyethylene glycol)] (DSPE‐PEG_5000_) was purchased from Avanti Polar Lipids. Cyanine5 (Cy5, 95+%) was purchased from Lumiprobe. The murine melanoma cell line (B16F10) and human cervical cancer cell line (HeLa) were obtained from ATCC. Dulbecco's Modified Eagle Medium (DMEM), PBS, FBS, trypsin‐ethylenediaminetetraacetic acid (trypsin‐EDTA, 0.25%), and antibiotics (10 000 Units/mL penicillin and 10 000 µg mL^−1^ streptomycin) were purchased from Gibco Life Technologies. Matrigel matrix was purchased from Corning. Primary antibodies: anti‐BRD4 (Bethyl Laboratories, A301‐985A100), dilution 1:1000 for western blot and immunofluorescence staining, 1:200 for IHC staining; anti‐c‐Myc (Abcam, ab32072), dilution 1:1000 for western blot assay and 1:200 for IHC staining; anti‐*β*‐Actin (Cell Signaling Technology, #8457), dilution 1:1000 for western blot. Secondary antibody: Alexa Fluor 568 Goat‐anti Rabbit IgG (Abcam, ab175471), dilution 1:1000 for immunofluorescence staining; anti‐rabbit horseradish peroxidase (HRP)‐conjugated secondary antibodies (Cell Signaling Technology, #7074S), dilution 1:2000.

### Synthesis and Characterization of PDSA Polymer

The PDSA polymer was synthesized by a one‐step polycondensation reaction according to the previous method. Briefly, (H‐Cys‐OMe)2.2HCl (10 mmol) and triethylamine (15 mmol) were dissolved in 20 mL of DMSO. Then, adipoyl chloride dissolved in 20 mL of DMSO was added dropwise into the solution under stirring. Afterward, the solution was stirred for 1 h to obtain a uniform mixture. Finally, the brown‐yellow PDSA precipitation was obtained by adding ice‐cold diethyl ether followed by drying under a vacuum for 48 h. The ^1^H NMR spectrum of the synthesized PDSA suspended in DMSO‐d^6^ was characterized by using a Mercury VX‐300 spectrometer at 400 MHz.

### Preparation and Characterization of ARV@PDSA

PDSA polymer (2.5 mg) and DSPE‐PEG_5000_ (0.5 mg) were first dissolved in DMF (60 µL) followed by mixing with 40 µL of DMSO containing 0.2 mg of ARV‐771 to form a homogenous solution. Then, 1.9 mL of deionized H_2_O was added to the mixture under vigorous stirring (1200 rpm). The synthesized nanoparticles were collected through centrifugation (3500 rpm) by centrifuge tubes with Amicon Ultra‐15 Centrifugal Filter (MWCO: 100 KD, Millipore). The pellet was washed with deionized H_2_O three times to remove the residual DMF, DMSO, and unloaded ARV‐771. The obtained ARV‐771‐loaded PDSA nanoparticles (ARV@PDSA) were finally dispersed in 2 mL of PBS buffer (pH 7.4) and stored at 4 °C for further use. All the procedures for the preparation of fluorescence dye (Cy5 or NileRed)‐labeled PDSA nanoparticles (Cy5@PDSA or NileRed@PDSA) were the same as that of ARV@PDSA except for the cargos loaded in the PDSA nanoparticles.

The DLS size and zeta potential values of PDSA and ARV@PDSA nanoparticles were measured by a laser particle analyzer (ZetaPALS, Brookhaven Instruments) at room temperature. The morphology and size of ARV@PDSA nanoparticles were characterized by TEM (Tecnai G2 Spirit BioTWIN). The redox responsiveness of ARV@PDSA was also characterized by DLS and TEM after incubation with DTT for different periods of time. The loading capacity and loading efficiency of ARV‐771 were measured by HPLC (Agilent 1260 Infinity II) equipped with a C‐18 column and UV detector set at 259 nm. The peak elution time of ARV‐771 was about 6.9 min using a mobile phase constituted of deionized H_2_O: acetonitrile (60:40, v/v) at a flow rate of 1 mL min^−1^.

The release of ARV‐771 from ARV@PDSA was carried out in PBS solutions containing 0.1 wt% tween 80 with different concentrations of DTT (0, 1, and 10 mm). In general, ARV@PDSA (0.5 mg) dispersed in PBS solutions (10 mm, pH 7.4, 0.1 wt% tween 80) containing 0, 1, or 10 mm DTT was added to 2 mL Eppendorf tubes and placed in a 37 °C incubator with shaking (120 rpm). At the pre‐determined time points, the samples were centrifuged at 11 000 g for 10 min, and the supernatants were collected to quantify the release efficiency of ARV‐771 by HPLC. Finally, the nanoparticle pellets were re‐dispersed in the same volume of the fresh‐release medium.

The loading capacity of ARV‐771 was defined as (mass of loaded ARV‐771/ mass of PDSA) × 100%) and the loading efficiency was defined as (mass of loaded ARV‐771/ mass of total ARV‐771) × 100%).

### Cell Culture and Cellular Internalization of Nanoparticles

Murine melanoma cells (B16F10) and human cervical cancer cells (HeLa) were cultured in DMEM supplemented with 10% FBS and 100 U mL^−1^ penicillin and 100 mg mL^−1^ streptomycin in a humidity‐controlled incubator at 37 °C with 5% CO_2_. The medium was changed every other day and the cells were sub‐cultured when they reached 90% of confluence.

B16F10 or HeLa cells were seeded in 24‐well plates with glass coverslips at a density of 4 × 10^4^ cells per well. After 24 h of attachment, the cells were incubated in 2 mL of fresh DMEM supplemented with 10% FBS and 100 U mL^−1^ of penicillin, and 100 mg mL^−1^ of streptomycin containing NileRed@PDSA at a NileRed concentration of 0.1 µg mL^−1^. After incubation for different periods of time, the cells were fixed with 4% paraformaldehyde (PFA) for 10 min, and the nuclei were strained with DAPI (10 µg mL^−1^) for 10 min at room temperature. In addition, the cytoskeleton was outlined by Alexa Fluor 488 phalloidin (Thermo Fisher Scientific, A12379), a high‐affinity probe to filamentous actin. The uptake of nanoparticles was observed under a Carl Zeiss Microscope (Axiovert 200).

### Western Blot Assay

HeLa or B16F10 cells were seeded in 6‐well plates at a density of 4 × 10^5^ cells per well for 24 h. After the attachment, the cells were treated by free ARV‐771 or ARV@PDSA at various concentrations (10, 20, 50, 100, and 200 nm ARV‐771) for 24 h or at an ARV‐771 concentration of 100 nm for different periods of time (2, 4, 8, 12, and 24 h). Additionally, to compare the expression levels of BRD4 and c‐Myc before and after treatment with ARV‐771, the cells were treated with 50 nm of free ARV‐771 for 24 h. The untreated cells served as a control. Then, the cells were washed with ice‐cold PBS three times followed by lysed in a RIPA lysis buffer solution (Thermo Fisher Scientific, 89 900) supplemented with 1% protease inhibitor cocktail (Thermo Fisher Scientific, A32953) on ice for 15 min. Afterward, the protein lysates were collected by centrifugation, and the protein concentration in the supernatants was quantified by a Pierce BCA protein Assay kit (Thermo Fisher Scientific, 23 225). The lysates were reduced within 4× SDS loading buffer (Bio‐Rad Laboratories, 1 610 747) and denatured at 100 °C for 10 min. The proteins were separated by 8% SDS‐PAGE (Invitrogen, XP00085BOX) followed by transfer to a PVDF membrane. Then, the membrane was blocked with 5% of BSA in TBST (150 mm NaCl, 50 mm Tris‐HCl at pH 7.4, and 0.1% Tween 20) at room temperature for 1 h followed by incubation with various primary antibodies overnight at 4 °C. The membranes were washed thoroughly with TBST (three times, 5 min/time), and incubated with appropriate HRP‐conjugated secondary antibodies at room temperature for 1 h. Enhanced chemiluminescence (ECL) was used to detect the targeted protein expression. *β*‐actin served as an internal reference protein.

### Immunofluorescence Assay

B16F10 or HeLa cells were seeded in 24‐well plates with glass coverslips at a density of 4 × 10^4^ cells per well. After attachment for 24 h, the cells were treated by ARV@PDSA at various concentrations (50, 100, and 200 nm) for 24 h. Afterward, the cells were fixed with a PBS solution containing 4% PFA for 10 min, permeabilized in a PBS solution containing 0.1% Triton X‐100 for 10 min, blocked in 5% BAS PBST (PBS containing 0.1% Tween 20) at room temperature for 1 h, and incubated with anti‐BRD4 primary antibody at 4 °C for overnight. Then, the cells were washed with PBST (three times, 5 min/time) and incubated with Alexa Fluor 568 Goat‐anti Rabbit IgG at room temperature for 1 h in a dark environment. Subsequently, the nuclei were stained by Hoechst 33 342 and the cytoskeleton was stained by Alexa Fluor 488 phalloidin. Finally, the samples were mounted by Prolong Gold antifade mounting medium (Invitrogen, S36937). The protein expression was observed under a confocal laser scanning microscope (Olympus FV1000).

### In Vitro Cytotoxicity Assay

HeLa cells were seeded in 96‐well plates at a density of 1 × 10^4^ cells per well. After attachment for 24 h, the cells were treated with or without DEM (1 mm) or GSH‐OEt (5 mm) for 2 h,^[^ ^45^, ^46^ ^]^ followed by 200 µL of fresh medium containing free ARV‐771 and ARV@PDSA at a concentration of 1000 nm ARV‐771 for 24 h. In terms of concentration‐dependent cytotoxicity, the seeded HeLa cells or B16F10 cells were incubated with free ARV‐771 or ARV@PDSA at the concentrations of 0, 50, 100, 200, 500, and 1000 nm ARV‐771 for 24 h. Then, 10 µL of MTT solution (5 mg mL^−1^) was added to each well followed by incubation for another 4 h. Finally, the medium was replaced by 100 µL of DMSO. The absorbance of the DMSO solutions in each well was measured at 570 nm using a microplate reader (TECAN, Infinite M Plex). The relative cell viability was calculated by the following formula: cell viability = *Abs*_a_/*Abs*_b_ × 100%, where *Abs*_a_ is the absorbance of treated cells, and *Abs*_b_ is the absorbance of the untreated cells (control).

### Colony Formation Assay

HeLa and B16F10 cells were seeded in six‐well plates at a density of 1 × 10^3^ cells per well. After 24 h attachment, the cells were treated by free ARV‐771 or ARV@PDSA at three different concentrations of ARV‐771 (50, 100, and 200 nm). After another 10 days of culture, the medium was discarded, and the attached cells were washed twice with PBS and fixed with 4% PFA. Subsequently, the cells were stained with 1% crystal violet for 15 min and washed with tap water three times. Finally, the stained colony was dried for the photograph.

### Tumor Spheroid Assay

Briefly, HeLa cells were seeded in 96‐well ultra‐low‐attachment (ULA) plates (Corning, MA, USA) at a density of 3 × 10^3^ cells per well in 100 µL of culture medium. After observing the formation of spheroids, free ARV‐771 and ARV@PDSA were added to the culture medium at three different concentrations (50, 100, and 200 nm). The morphology of tumor spheroids was observed and imaged using a fluorescence microscope every day (EvosTM FL, ThermoFisher Scientific, MA, USA). The culture medium was carefully replaced by a fresh one containing different concentrations of drugs every three days.

### Cell Membrane Lipid Peroxidation Assay

HeLa cells were seeded in 6‐well plates at a density of 4 × 10^5^ cells per well. After 24 h attachment, the cells were treated by free ARV‐771 or ARV@PDSA at an ARV‐771 concentration of 1000 nm for another 24 h. Then, the cells were co‐incubated with C11‐BODIPY 581/591 (Thermo Fisher Scientific, D3861, 10 µm) and Hoechst 33 342 (8 µg mL^−1^) in PBS for 30 min. The stained cells were washed three times with PBS and imaged under an automated microscope (Evos FL Auto 2, Thermo Fisher Scientific) to visualize the reduced and oxidized fluorescent dye with the Texas Red filter (585/624 nm) and GFP filter (470/525 nm), respectively.

### Cell Cycle Assay

HeLa cells were seeded in 6‐well plates at a density of 4 × 10^5^ cells per well for 24 h. Following the attachment, the cells were treated by free ARV‐771 or ARV@PDSA at an ARV‐771 concentration of 1000 nm for another 24 h. Then, the cells were harvested with 0.25% trypsin, washed once with cold PBS, and placed in precooled 70% ethanol at −20 °C for 24 h for fixation. Then, the cells were washed with PBS once and incubated with propidium iodide (50 µg mL^−1^, Thermo Fisher Scientific, F10797) in a dark environment at room temperature for 30 min. Subsequently, the cells were analyzed using a flow cytometer (BD, LSRFortessa) and the data were analyzed using FlowJo software, version 8.8.4 (Tree star, Inc., Ashland, Or, USA).

### Animals

All the in vivo animal experiments were carried out in accordance with the National Institutes of Health (NIH) guidelines, and approved protocol by the Institutional Animal Care and Use Committees at Brigham and Women's Hospital (BWH) and Harvard Medical School (HMS) (Approval Number: 2020N000055). The animal studies were performed under strict pathogen‐free conditions in the animal facilities of BWH. Female C57BL/6 mice (4–5 weeks old) were purchased from Charles River Laboratories and female athymic nude mice (NU/J, *Foxn1^nu^*, 4–6 weeks old) were purchased from Jackson Laboratory.

### Tumor Model

To establish HeLa xenograft tumor model, 5 × 10^6^ HeLa cells suspended in 100 µL of mixture solution of Matrigel and PBS (volume: volume = 1:1) were subcutaneously injected into the right hind leg of nude mice. When the tumor volume reached 50–100 mm^3^, the mice were randomly divided into six different groups for further experiments.

To establish the B16F10 xenograft tumor model, 5 × 10^5^ B16F10 cells suspended in 100 µL of PBS were subcutaneously injected into the right hind leg of C57BL/6 mice. When the tumor volume reached 50–100 mm^3^, the mice were randomly divided into six different groups for further experiments. The tumor size was measured every other day by a digital caliber and was calculated by the following equation: tumor volume = width^2^ × length/2.

### In Vivo Biodistribution of Cy5@PDSA Nanoparticles

HeLa tumor‐bearing mice were administered with free Cy5, or Cy5@PDSA nanoparticles via the tail vein at a Cy5 dose of 0.5 mg kg^−1^. Then, the mice were anesthetized and imaged using a Syngene PXi imaging system (Synoptics Ltd.) 3, 6, 12, and 24 h post‐administration. After 24 h, the mice were sacrificed, and their tumor and main organs, including heart, liver, spleen, lung, and kidneys, were harvested for ex vivo imaging to study the biodistribution of the administered nanoparticles.

### In Vivo Antitumor Experiment

HeLa or B16F10 tumor‐bearing mice (tumor volume ≈100 mm^3^) were randomly divided into six groups (*n* = 4 or 5): I) control (PBS); II) PDSA NPs; III) ARV@PDSA (10 mg kg^−1^); IV) ARV@PDSA (3 mg kg^−1^); V) free ARV‐771 (10 mg kg^−1^); and VI) free ARV‐771 (3 mg kg^−1^). For groups II, III, and IV, the mice were given intravenously 200 µL of PBS containing PDAS or ARV@PDSA. For groups V and VI, the mice were given intravenously 200 µL of the mixture of 0.5% DMSO + 5% ethanol + 0.5% Tween 80 + ddH_2_O containing ARV‐771) every other day five times (B16F10 tumor model) or seven times (HeLa tumor model).^[^ ^13^ ^]^ The tumor size and body weight of the mice were measured every other day over a period of 12 days for HeLa tumors and 8 days for B16F10 tumors. One day after the last administration, all the mice were sacrificed, and the tumors were collected, weighed, and photographed. Subsequently, the tumors were cut into two parts, one part was stored at −80 °C for the western blot assay, and the other part was fixed in 4% PFA for IHC. The main organs, including the heart, liver, spleen, lung, and kidney, were harvested for H&E staining to evaluate the biocompatibility and biosafety of all the treatments.

### Statistical Analysis

All data were presented as the mean ± standard deviations (SD) or means ± standard error of mean (s.e.m.) from at least three repeated experiments. Differences among the groups were determined using a two‐tailed Student's *t*‐test, one‐way ANOVA with a Tukey post hoc test, or two‐way ANOVA with Sidak's test using GraphPad Prism 7.0. *p* < 0.05 is considered significantly different, the significance levels are *p < 0.05, **p < 0.01, ***p < 0.001, and ns denotes not significant.

## Conflict of Interest

The authors declare no conflict of interest.

## Supporting information

Supporting Information

## Data Availability

The data that support the findings of this study are available in the supplementary material of this article.

## References

[1] K. M. Sakamoto , K. B. Kim , A. Kumagai , F. Mercurio , C. M. Crews , R. J. Deshaies , Proc. Natl. Acad. Sci. U. S. A. 2001, 98, 8554. 11438690 10.1073/pnas.141230798 PMC37474

[2] A. C. Lai , C. M. Crews , Nat. Rev. Drug Discovery 2017, 16, 101. 27885283 10.1038/nrd.2016.211 PMC5684876

[3] M. Békés , D. R. Langley , C. M. Crews , Nat. Rev. Drug Discovery 2022, 21, 181. 35042991 10.1038/s41573-021-00371-6 PMC8765495

[4] X. Sun , H. Gao , Y. Yang , M. He , Y. Wu , Y. Song , Y. Tong , Y. Rao , Signal Transduct. Target. Ther. 2019, 4, 64. 31885879 10.1038/s41392-019-0101-6 PMC6927964

[5] D. P. Bondeson , A. Mares , I. E. Smith , E. Ko , S. Campos , A. H. Miah , K. E. Mulholland , N. Routly , D. L. Buckley , J. L. Gustafson , Nat. Chem. Biol. 2015, 11, 611. 26075522 10.1038/nchembio.1858 PMC4629852

[6] H. Gao , X. Sun , Y. Rao , ACS Med. Chem. Lett. 2020, 11, 237. 32184950 10.1021/acsmedchemlett.9b00597 PMC7073876

[7] US National Library of Medicine, ClinicalTrials.gov 2021, 3, https://clinicaltrials.gov/ct2/show/NCT03888612.

[8] US National Library of Medicine. ClinicalTrials.gov 2022, 8, https://clinicaltrials.gov/ct2/show/NCT04072952.

[9] V. G. Klein , C. E. Townsend , A. Testa , M. Zengerle , C. Maniaci , S. J. Hughes , K.‐H. Chan , A. Ciulli , R. S. Lokey , ACS Med. Chem. Lett. 2020, 11, 1732. 32939229 10.1021/acsmedchemlett.0c00265 PMC7488288

[10] K. Raina , J. Lu , Y. Qian , M. Altieri , D. Gordon , A. M. K. Rossi , J. Wang , X. Chen , H. Dong , K. Siu , Proc. Natl. Acad. Sci. U. S. A. 2016, 113, 7124. 27274052 10.1073/pnas.1521738113 PMC4932933

[11] A. D. Buhimschi , H. A. Armstrong , M. Toure , S. Jaime‐Figueroa , T. L. Chen , A. M. Lehman , J. A. Woyach , A. J. Johnson , J. C. Byrd , C. M. Crews , Biochemistry 2018, 57, 3564. 29851337 10.1021/acs.biochem.8b00391

[12] G. F. Watt , P. Scott‐Stevens , L. Gaohua , Drug Discovery Today Technol. 2019, 31, 69. 10.1016/j.ddtec.2019.02.005 31200862

[13] S. He , F. Gao , J. Ma , H. Ma , G. Dong , C. Sheng , Angew. Chem., Int. Ed. 2021, 60, 23299. 10.1002/anie.202107347 34240523

[14] J. Shao , Y. Yan , D. Ding , D. Wang , Y. He , Y. Pan , W. Yan , A. Kharbanda , H. y. Li , H. Huang , Adv. Sci. 2021, 8, 2102555. 10.1002/advs.202102555 PMC8529430 34397171

[15] J. Liu , H. Chen , Y. Liu , Y. Shen , F. Meng , H. U. Kaniskan , J. Jin , W. Wei , J. Am. Chem. Soc. 2021, 143, 7380. 33970635 10.1021/jacs.1c00451 PMC8219215

[16] W. Chen , M. Schilperoort , Y. Cao , J. Shi , I. Tabas , W. Tao , Nat. Rev. Cardiol. 2022, 19, 228. 34759324 10.1038/s41569-021-00629-x PMC8580169

[17] W. Tao , N. Kong , X. Ji , Y. Zhang , A. Sharma , J. Ouyang , B. Qi , J. Wang , N. Xie , C. Kang , Chem. Soc. Rev. 2019, 48, 2891. 31120049 10.1039/c8cs00823j

[18] C. Liu , J. Shin , S. Son , Y. Choe , N. Farokhzad , Z. Tang , Y. Xiao , N. Kong , T. Xie , J. S. Kim , Chem. Soc. Rev. 2021, 50, 2260. 33367452 10.1039/d0cs01175d

[19] J. Shi , P. W. Kantoff , R. Wooster , O. C. Farokhzad , Nat. Rev. Cancer 2017, 17, 20. 27834398 10.1038/nrc.2016.108 PMC5575742

[20] C. Zhang , J. Huang , Z. Zeng , S. He , P. Cheng , J. Li , K. Pu , Nat. Commun. 2022, 13, 3468. 35710545 10.1038/s41467-022-31044-6 PMC9203767

[21] C. Zhang , K. Pu , Chem. Soc. Rev. 2020, 49, 4234. 32452475 10.1039/c9cs00773c

[22] J. Chen , M. Qiu , F. Ma , L. Yang , Z. Glass , Q. Xu , J. Controlled Release 2021, 330, 1244. 10.1016/j.jconrel.2020.11.032 PMC7906926 33234362

[23] A. Saraswat , M. Patki , Y. Fu , S. Barot , V. V. Dukhande , K. Patel , Nanomedicine 2020, 15, 1761. 32698663 10.2217/nnm-2020-0156

[24] J. Y. Lin , H. J. Liu , Y. Wu , J. M. Jin , Y. D. Zhou , H. Zhang , D. G. Nagle , H. Z. Chen , W. D. Zhang , X. Luan , Small 2023, 2207778.

[25] J. Gao , B. Hou , Q. Zhu , L. Yang , X. Jiang , Z. Zou , X. Li , T. Xu , M. Zheng , Y.‐H. Chen , Z. Xu , H. Xu , H. Yu , Nat. Commun. 2022, 13, 4318. 35882867 10.1038/s41467-022-32050-4 PMC9325692

[26] C. Zhang , Z. Zeng , D. Cui , S. He , Y. Jiang , J. Li , J. Huang , K. Pu , Nat. Commun. 2021, 12, 2934. 34006860 10.1038/s41467-021-23194-w PMC8131624

[27] C. Zhang , S. He , Z. Zeng , P. Cheng , K. Pu , Angew. Chem., Int. Ed. 2022, 61, e202114957. 10.1002/anie.202114957 34927316

[28] X. Ling , J. Tu , J. Wang , A. Shajii , N. Kong , C. Feng , Y. Zhang , M. Yu , T. Xie , Z. Bharwani , ACS Nano 2018, 13, 357. 30485068 10.1021/acsnano.8b06400 PMC7049173

[29] A. Biroccio , B. Benassi , G. Filomeni , S. Amodei , S. Marchini , G. Chiorino , G. Rotilio , G. Zupi , M. R. Ciriolo , J. Biol. Chem. 2002, 277, 43763. 12226097 10.1074/jbc.M207684200

[30] J. Wu , L. Zhao , X. Xu , N. Bertrand , W. I. Choi , B. Yameen , J. Shi , V. Shah , M. Mulvale , J. L. MacLean , Angew. Chem., Int. Ed. 2015, 54, 9218. 10.1002/anie.201503863 26119453

[31] N. Kong , W. Tao , X. Ling , J. Wang , Y. Xiao , S. Shi , X. Ji , A. Shajii , S. T. Gan , N. Y. Kim , Sci. Transl. Med. 2019, 11, eaaw1565. 31852795 10.1126/scitranslmed.aaw1565 PMC7024563

[32] P. Ottis , C. M. Crews , ACS Chem. Biol. 2017, 12, 892. 28263557 10.1021/acschembio.6b01068

[33] S. Sui , J. Zhang , S. Xu , Q. Wang , P. Wang , D. Pang , Cell Death Dis. 2019, 10, 331. 30988278 10.1038/s41419-019-1564-7 PMC6465411

[34] Q. Shao , A. Kannan , Z. Lin , B. C. Stack , J. Y. Suen , L. Gao , Cancer Res. 2014, 74, 7090. 25277525 10.1158/0008-5472.CAN-14-0305 PMC4322674

[35] N. A. Franken , H. M. Rodermond , J. Stap , J. Haveman , C. Van Bree , Nat. Protoc. 2006, 1, 2315. 17406473 10.1038/nprot.2006.339

[36] H.‐j. Liu , J. Wang , M. Wang , Y. Wang , S. Shi , X. Hu , Q. Zhang , D. Fan , P. Xu , Adv. Funct. Mater. 2021, 31, 2100262. 10.1002/adfm.202010556 PMC8376022 34421476

[37] J. Chen , J. Ding , Y. Wang , J. Cheng , S. Ji , X. Zhuang , X. Chen , Adv. Mater. 2017, 29, 1701170. 10.1002/adma.201701170 28632302

[38] X. L. Liu , X. Dong , S. C. Yang , X. Lai , H. J. Liu , Y. Gao , H. Y. Feng , M. H. Zhu , Y. Yuan , Q. Lu , Adv. Sci. 2021, 8, 2003679. 10.1002/advs.202003679 PMC8061387 33898179

[39] U. Prabhakar , H. Maeda , R. K. Jain , E. M. Sevick‐Muraca , W. Zamboni , O. C. Farokhzad , S. T. Barry , A. Gabizon , P. Grodzinski , D. C. Blakey , Cancer Res. 2013, 73, 2412. 23423979 10.1158/0008-5472.CAN-12-4561 PMC3916009

[40] R. I. Troup , C. Fallan , M. G. Baud , Explor. Targeted Anti‐Tumor Ther. 2020, 1, 273. 10.37349/etat.2020.00018 PMC9400730 36046485

[41] J. Liu , Z. Zhao , N. Qiu , Q. Zhou , G. Wang , H. Jiang , Y. Piao , Z. Zhou , J. Tang , Y. Shen , Nat. Commun. 2021, 12, 2425. 33893275 10.1038/s41467-021-22407-6 PMC8065121

[42] X. Ling , X. Chen , I. A. Riddell , W. Tao , J. Wang , G. Hollett , S. J. Lippard , O. C. Farokhzad , J. Shi , J. Wu , Nano Lett. 2018, 18, 4618. 29902013 10.1021/acs.nanolett.8b01924 PMC6271432

[43] Y. Xiao , Z. Tang , X. Huang , W. Chen , J. Zhou , H. Liu , C. Liu , N. Kong , W. Tao , Chem. Soc. Rev. 2022, 51, 3828. 35437544 10.1039/d1cs00617g

[44] X. Huang , C. Liu , N. Kong , Y. Xiao , A. Yurdagul , I. Tabas , W. Tao , Nat. Protoc. 2022, 17, 748. 35121853 10.1038/s41596-021-00665-4 PMC9734002

[45] H. J. Liu , X. Luan , H. Y. Feng , X. Dong , S. C. Yang , Z. J. Chen , Q. Y. Cai , Q. Lu , Y. Zhang , P. Sun , Adv. Funct. Mater. 2018, 28, 1801118.

[46] K. Li , W. Dong , Y. Miao , Q. Liu , L. Qiu , J. Lin , J. Photochem. Photobiol., B 2021, 215, 112107. 33401190 10.1016/j.jphotobiol.2020.112107

