# Supplementary material for: Glutathione‐Scavenging Nanoparticle‐Mediated PROTACs Delivery for Targeted Protein Degradation and Amplified Antitumor Effects
Source: Adv Sci (Weinh). 2023 Apr 17;10(16):2207439. doi: 10.1002/advs.202207439 (PMC10238184; doi:10.1002/advs.202207439)
Supplement: Supplementary file 1 — Supporting Information [file ADVS-10-2207439-s001.pdf]

ADVANCED SCIENCE
Open Access

Supporting Information

for *Adv. Sci.*, DOI 10.1002/advs.202207439

Glutathione-Scavenging Nanoparticle-Mediated PROTACs Delivery for Targeted Protein Degradation and Amplified Antitumor Effects

*Hai-Jun Liu, Wei Chen, Gongwei Wu, Jun Zhou, Chuang Liu, Zhongmin Tang, Xiangang Huang, Jingjing Gao, Yufen Xiao, Na Kong, Nitin Joshi, Yihai Cao, Reza Abdi and Wei Tao**

Supporting Information

Glutathione-scavenging nanoparticle-mediated PROTACs delivery for targeted protein degradation and amplified antitumor effects

*Hai-Jun Liu, Wei Chen, Gongwei Wu, Jun Zhou, Chuang Liu, Zhongmin Tang, Xiangang Huang, Jingjing Gao, Yufen Xiao, Na Kong, Nitin Joshi, Yihai Cao, Reza Abdi, Wei Tao**

Dr. H. Liu, Dr. W. Chen, Dr. J. Zhou, Dr. C. Liu, Dr. Z. Tang, Dr. X. Huang, Dr. J. Gao, Dr. Y. Xiao, Dr. N. Kong, Prof. N. Joshi, Prof. W. Tao*
Center for Nanomedicine and Department of Anesthesiology, Brigham and Women's Hospital, Harvard Medical School, Boston, MA 02115, USA
E-mail: wtao@bwh.harvard.edu (Wei Tao)

Dr. H. Liu, Prof. R. Abdi
Transplantation Research Center, Renal Division, Brigham and Women's Hospital, Harvard Medical School, Boston, MA 02115, USA

Dr. G. Wu
Department of Medical Oncology, Dana-Farber Cancer Institute, Harvard Medical School, Boston, MA 02215, USA

Prof. Y. Cao
Department of Microbiology, Tumor and Cell Biology, Karolinska Institute, Stockholm 171 77, Sweden

H.-J.L., W.C., and G.W. contributed equally to this work.

Supplementary Figure

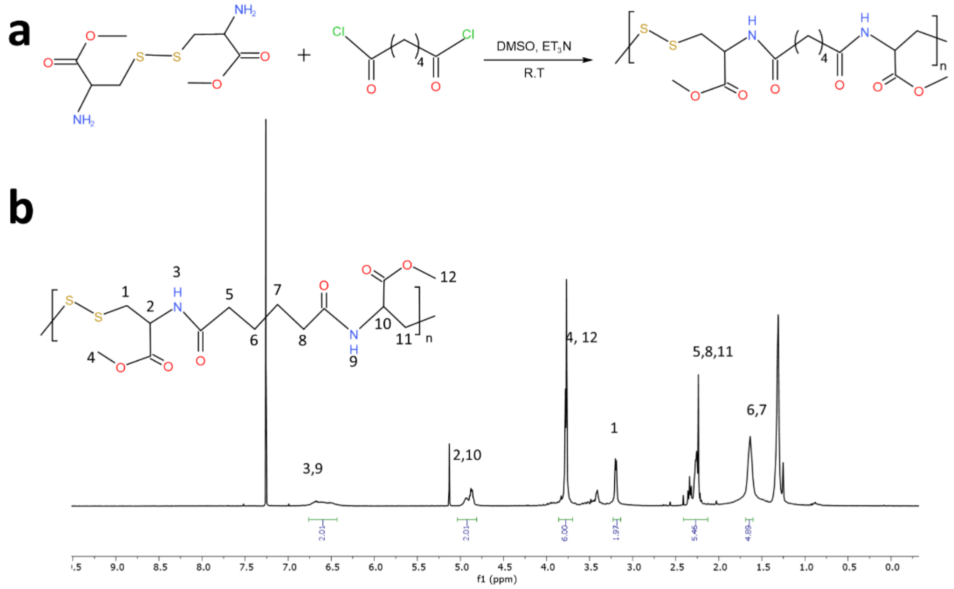

Fig. S1. Synthesis and characterization of PDSA polymer. (a) Synthesis route and (b) ^{1}H NMR spectrum of PDSA polymer. DMSO, dimethyl sulfoxide; Et_3N, triethylamine; R.T., room temperature.

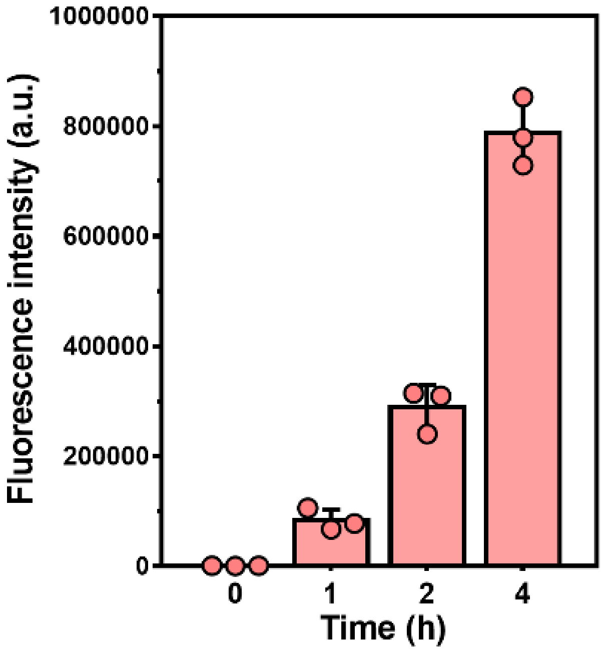

Fig. S2. Cellular uptake analysis of PDSA nanoparticles in HeLa cells. Quantitation of intracellular fluorescence intensity of Nile Red-labeled PDSA nanoparticles in HeLa cells from Figure 2b. Data are shown as mean ± s.e.m (n = 3).

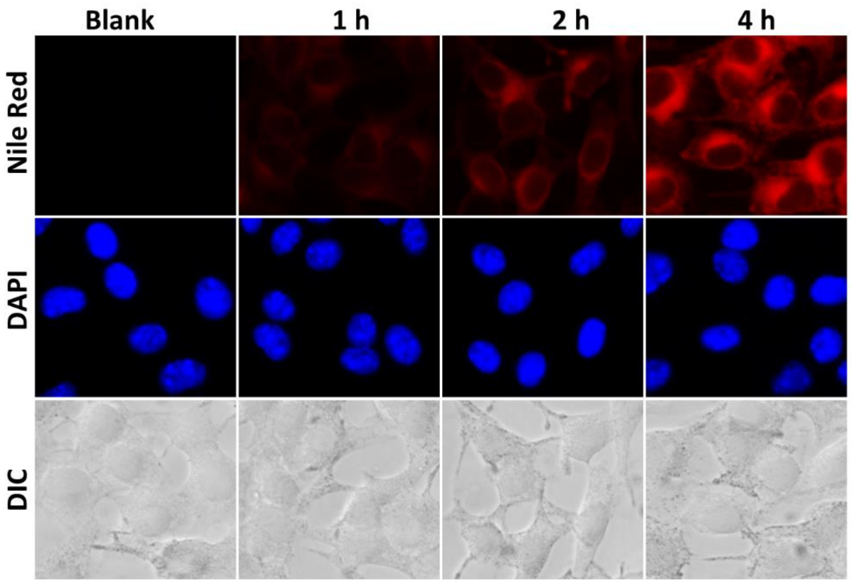

Fig. S3. Cellular uptake analysis of PDSA nanoparticles. Fluorescence microscope images of uptake of Nile Red-labeled PDSA nanoparticles in B16F10 cells for different periods of time.

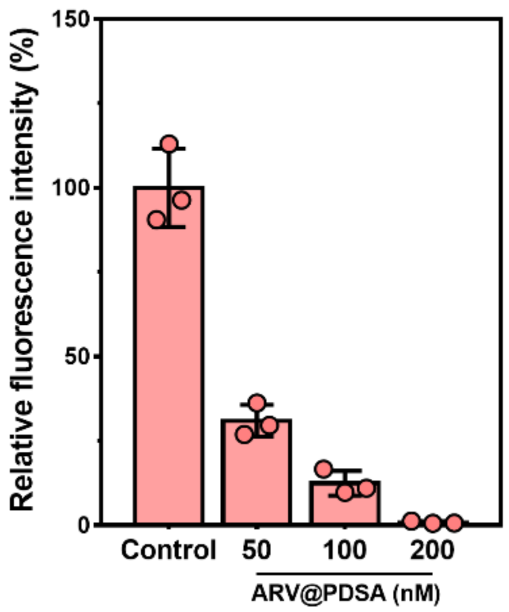

Fig. S4. Concentration-dependent targeted degradation of BRD4 in HeLa cells. Relative fluorescence intensity of BRD4 after the treatment with different concentration of ARV@PDSA from Figure 2e. Data are shown as mean ± s.e.m. (n = 3).

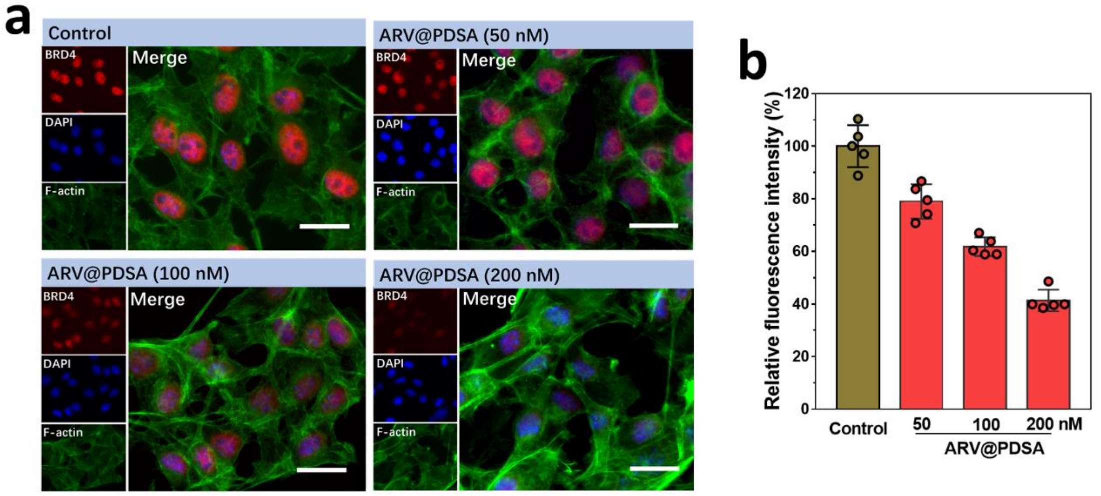

Fig. S5. Targeted degradation of BRD4. (a) Representative immunofluorescence microscope images of BRD4 protein in B16F10 cells after the treatment with various concentrations of ARV@PDSA for 24 h. Scale bars, 25 μm. (b) Relative fluorescence intensity of BRD4 after various treatments from (a). Data are shown as mean ± SD (n = 5).

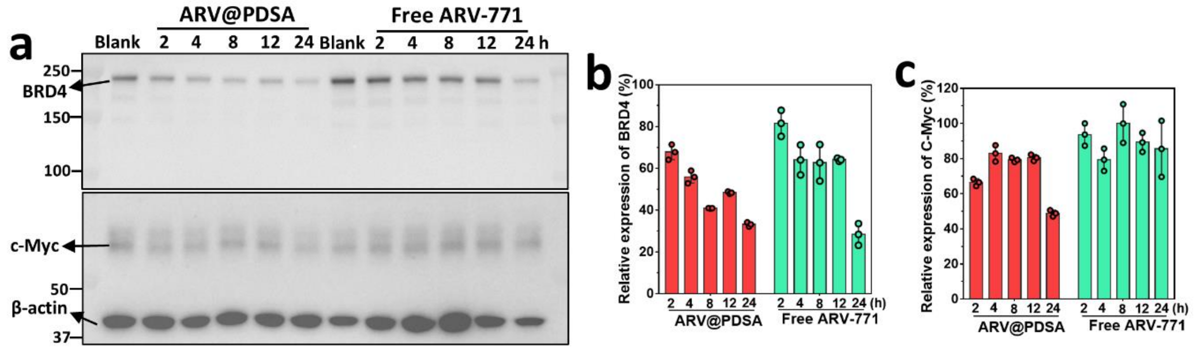

Fig. S6. Targeted degradation of BRD4 and downregulation of c-Myc. (a) Western blot assay of the expression levels of BRD4 and c-Myc in B16F10 cells after incubation with ARV@PDSA and free ARV-771 at the ARV-771 concentration of 100 nM for different periods of time. Quantification of the band intensity of (b) BRD4 and (c) c-Myc from (a). Data are shown as mean ± SD (n = 3).

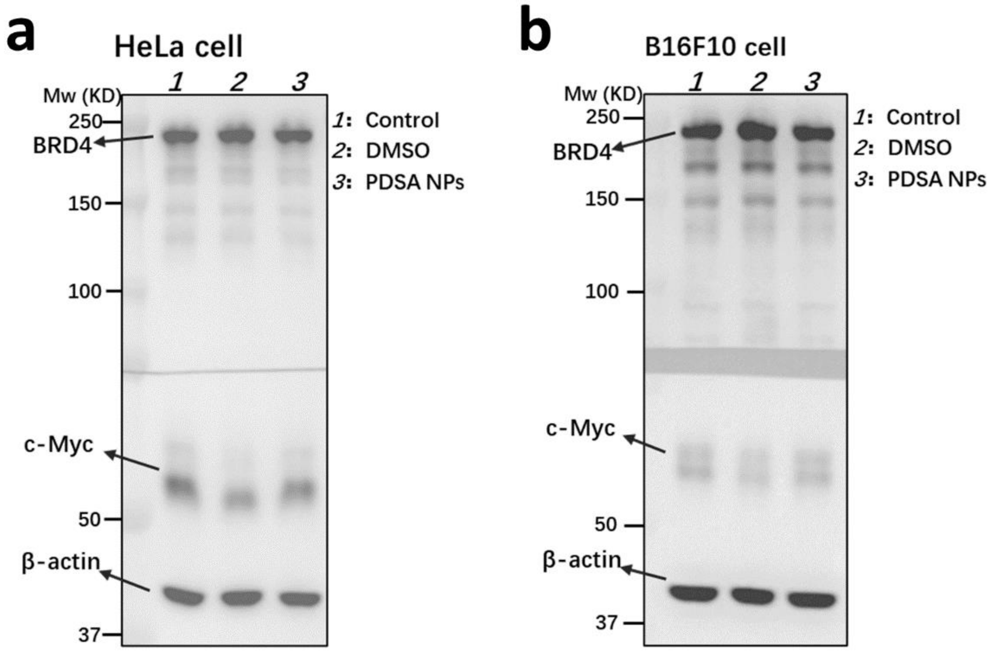

Fig. S7. Expression of BRD4 and c-Myc in DMSO and PDSA control groups. Western blot assay of the effect of dimethyl sulfoxide (DMSO) and PDSA nanoparticles on the expression of BRD4 and c-Myc in HeLa and B16F10 cells.

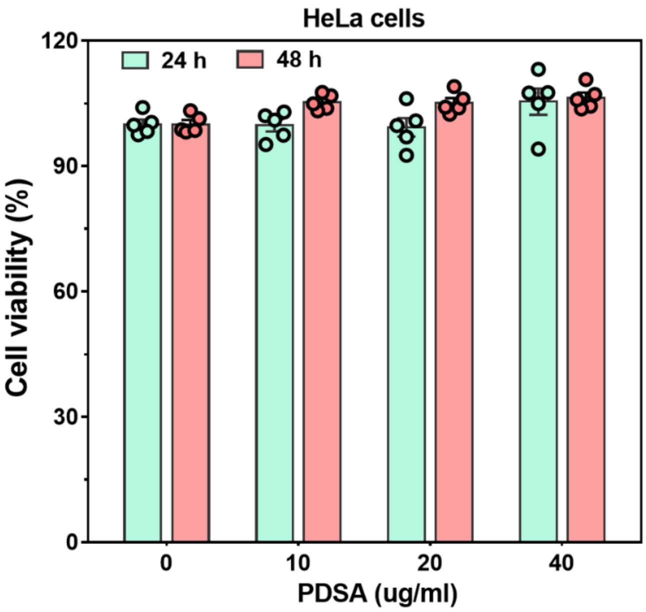

Fig. S8. *In vitro* biocompatibility of PDSA nanoparticles. Viability of HeLa cells after incubation with different concentrations of PDSA nanoparticles for 24 h and 48 h. Data are shown as mean ± s.e.m. (n = 5).

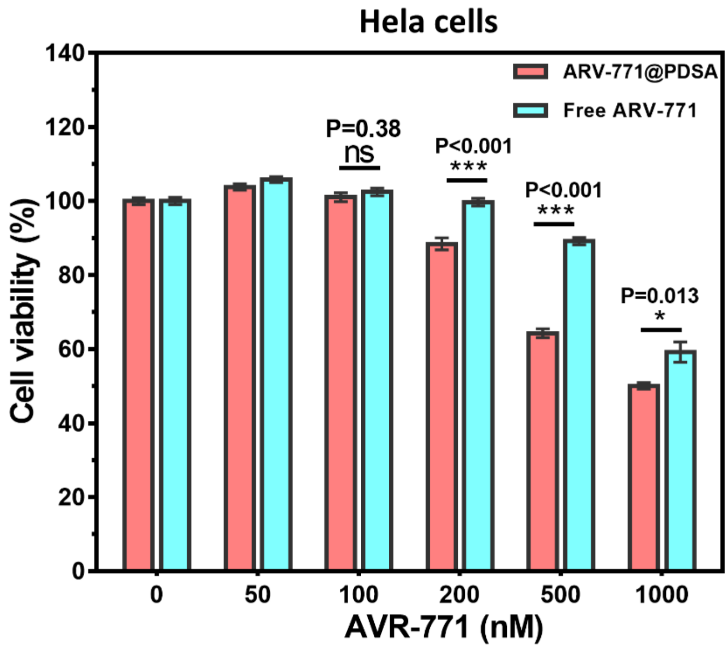

Fig. S9. Assessment of the *in vitro* anti-cancer effect of ARV@PDSA on the viability of HeLa cells. Viability of HeLa cells after the treatment with different concentrations of ARV@PDSA or free ARV-771 for 24 h. Data are shown as mean ± s.e.m. ($n = 5$). Statistical significance was determined by a two-tailed student's t-test (*$P < 0.05$, **$P < 0.01$, ***$P < 0.001$, and ns denotes not significant).

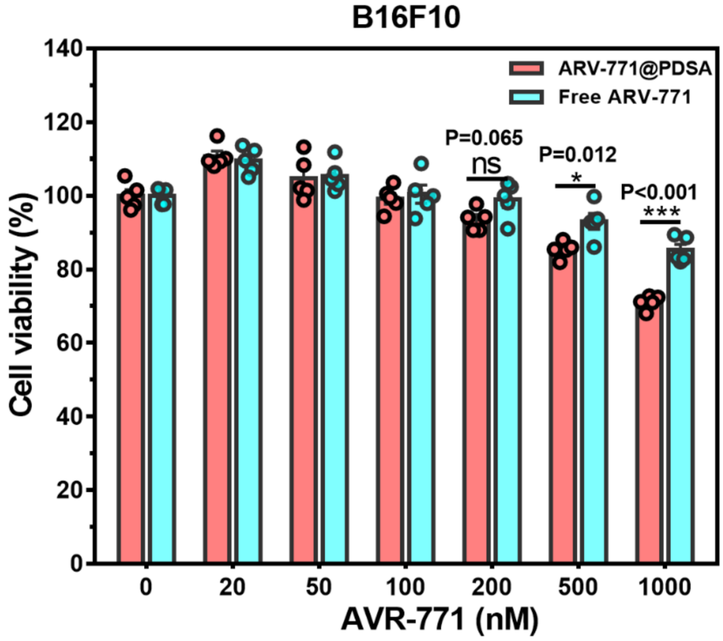

Fig. S10. Assessment of the *in vitro* anti-cancer effect of ARV@PDSA on the viability of B16F10 cells. Viability of B16F10 cells after the treatment with different concentrations of ARV@PDSA and free ARV-771 for 24 h. Data are shown as mean ± s.e.m. (n = 5). Statistical significance was determined by a two-tailed student's t-test (*P < 0.05, **P < 0.01 and ***P < 0.001, and ns denotes not significant).

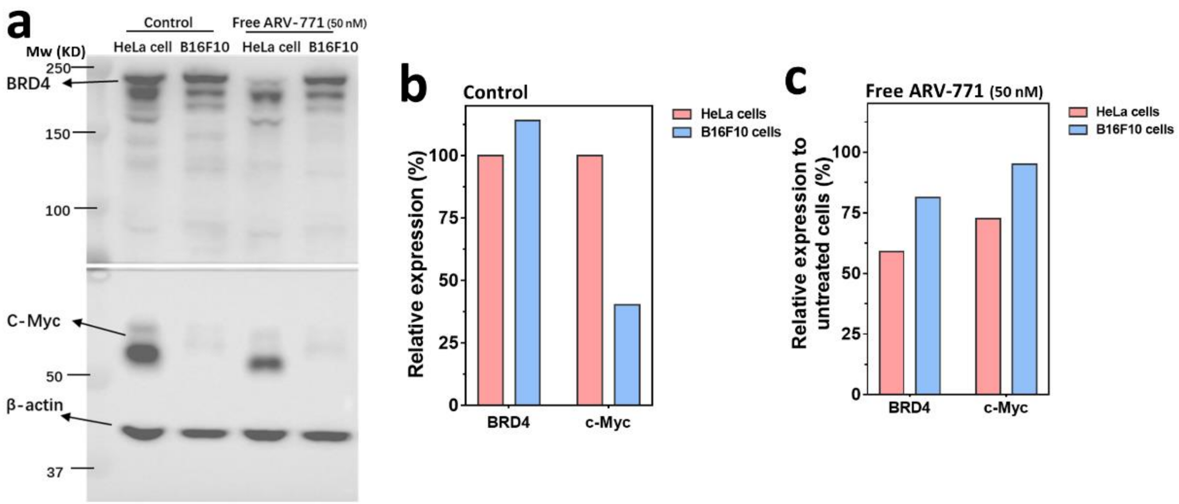

Fig. S11. Expression of BRD4 and c-Myc in HeLa and B16F10 cells. (a) Western blot analysis of the basal expression level of BRD4 and c-Myc in HeLa cells and B16F10 cells, and their sensitivity to free ARV-771. Quantification analysis of band intensity of BRD4 and c-Myc from (a) showing their (b) basal expression level and (c) sensitivity to free ARV-771 in HeLa cells and B16F10 cells.

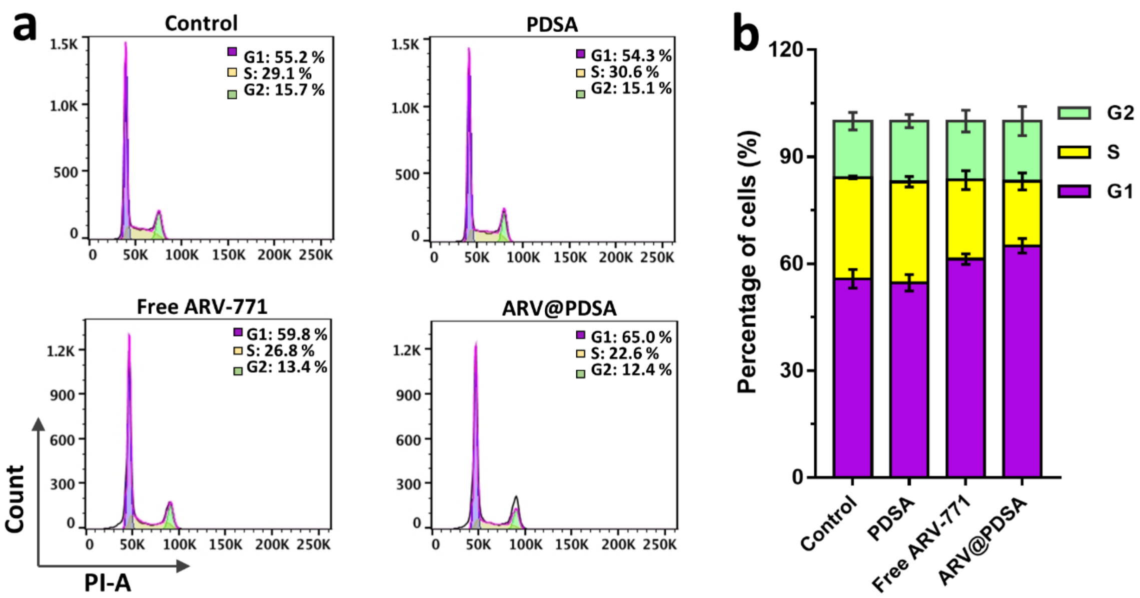

Fig. S12. *In vitro* cell cycle study. (a) Flow cytometry plots and (b) histograms of cell cycle distribution of HeLa cells after the treatment with ARV@PDSA and free ARV-771 at an ARV-771 concentration of 1000 nM for 24 h. Data are presented as mean ± SD (n = 3).

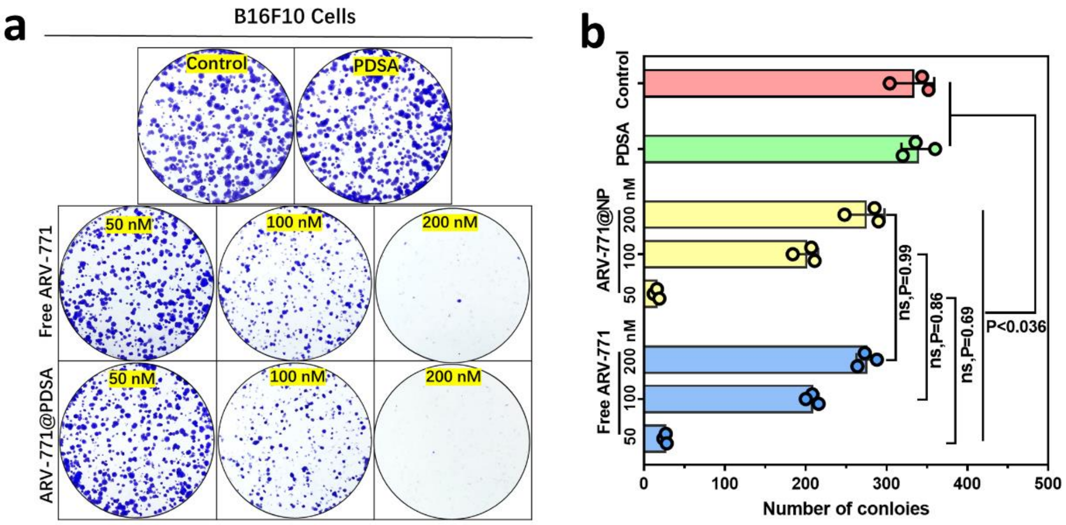

Fig. S13. *In vitro* anti-cancer effect of ARV@PDSA on B16F10 colony formation. (a) Representative microscope images of the colony formation assay of B16F10 cells stained with crystal violet after the treatment with ARV@PDSA and free ARV-771 at different concentrations. (b) Quantification of the number of colonies in various groups from (a). Data are shown as mean ± SD (n = 3), and analyzed by two-way ANOVA with Sidak's test. (*$P < 0.05$, **$P < 0.01$, ***$P < 0.001$, and ns denotes not significant).

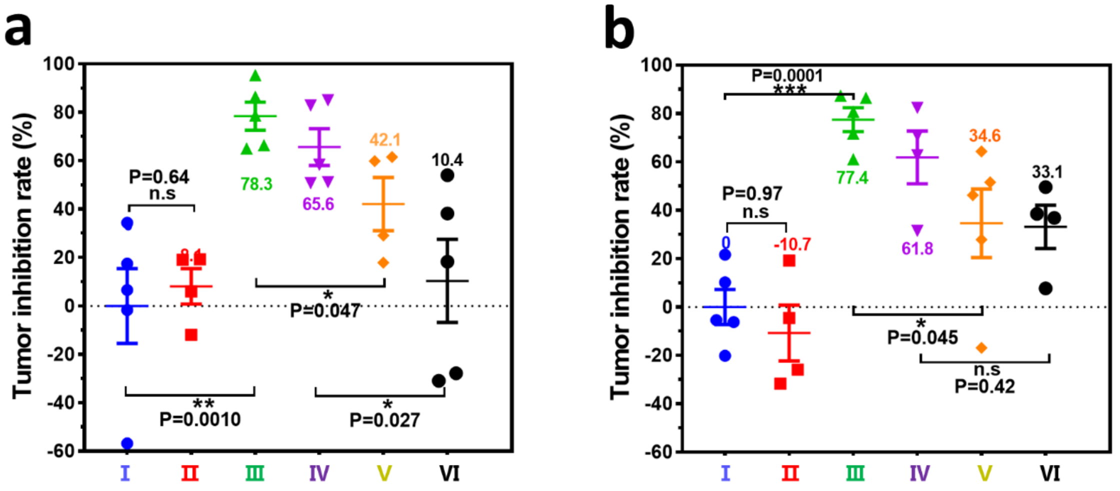

Fig. S14. *In vivo* tumor inhibition rate. Tumor inhibition rate of various treatment groups related to the control group (I) of (a) HeLa tumor-bearing athymic nude mice and (b) B16F10 tumor-bearing C57BL/6 mice. Data are shown as mean ± s.e.m. (n = 4 or 5), and analyzed by one-way ANOVA with a Tukey post hoc test. (*$P < 0.05$, **$P < 0.01$, ***$P < 0.001$, and ns denotes not significant). (I: Control, II: PDSA NPs, III: ARV@PDSA (10 mg/kg), IV: ARV@PDSA (3 mg/kg), V: Free ARV-771 (10 mg/kg), VI: Free ARV-771 (3 mg/kg)).

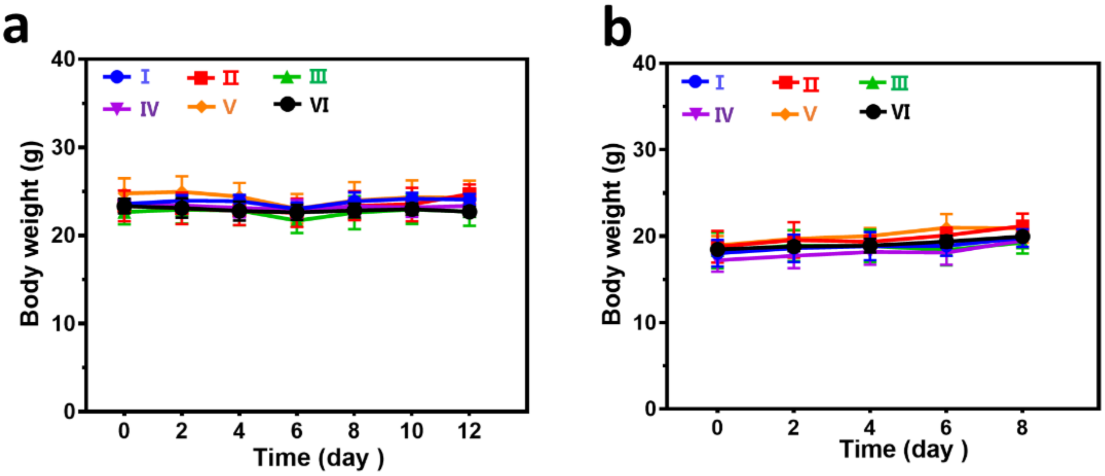

Fig. S15. Mouse body weight after receiving various treatments. Time-dependent body weights of (a) HeLa tumor-bearing athymic nude mice and (b) B16F10 tumor-bearing C57BL/6 mice of various treatment groups. Data are shown as mean ± s.e.m. (n = 4 or 5). (I: Control, II: PDSA NPs, III: ARV@PDSA (10 mg/kg), IV: ARV@PDSA (3 mg/kg), V: Free ARV-771 (10 mg/kg), VI: Free ARV-771 (3 mg/kg)).

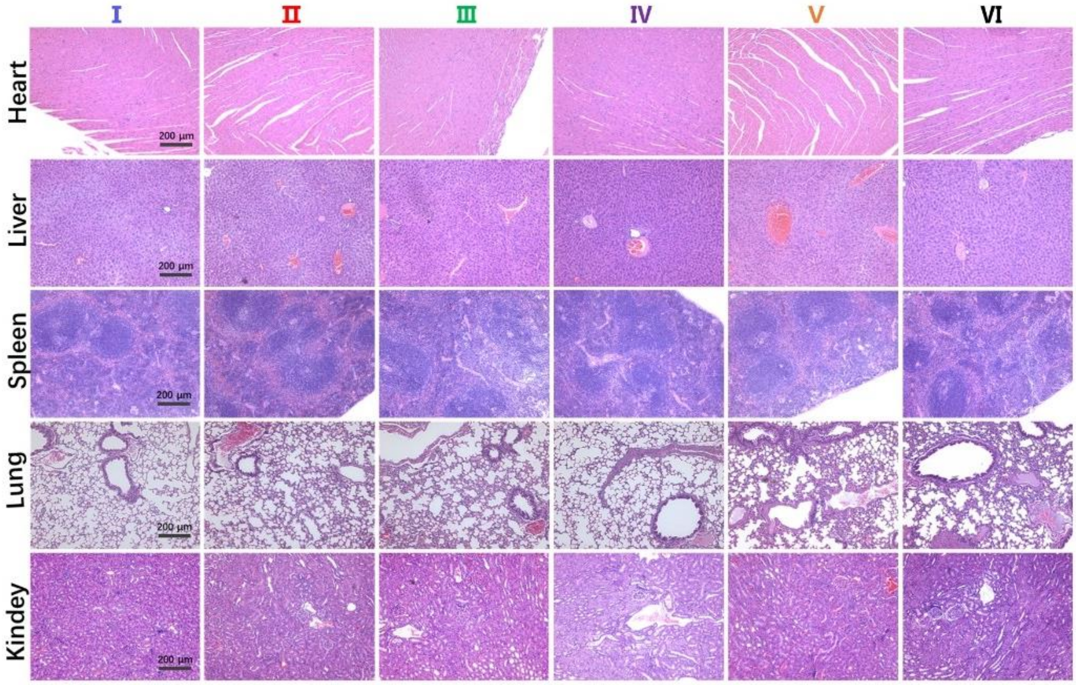

Fig. S16. Hematoxylin and Eosin (H&E) staining assay of HeLa tumor-bearing mice. H&E staining assay of the main organs (heart, liver, spleen, lung, and kidney) of HeLa tumor-bearing athymic nude mice in different treatment groups. (I: Control, II: PDSA NPs, III: ARV@PDSA (10 mg/kg), IV: ARV@PDSA (3 mg/kg), V: Free ARV-771 (10 mg/kg), VI: Free ARV-771 (3 mg/kg)).

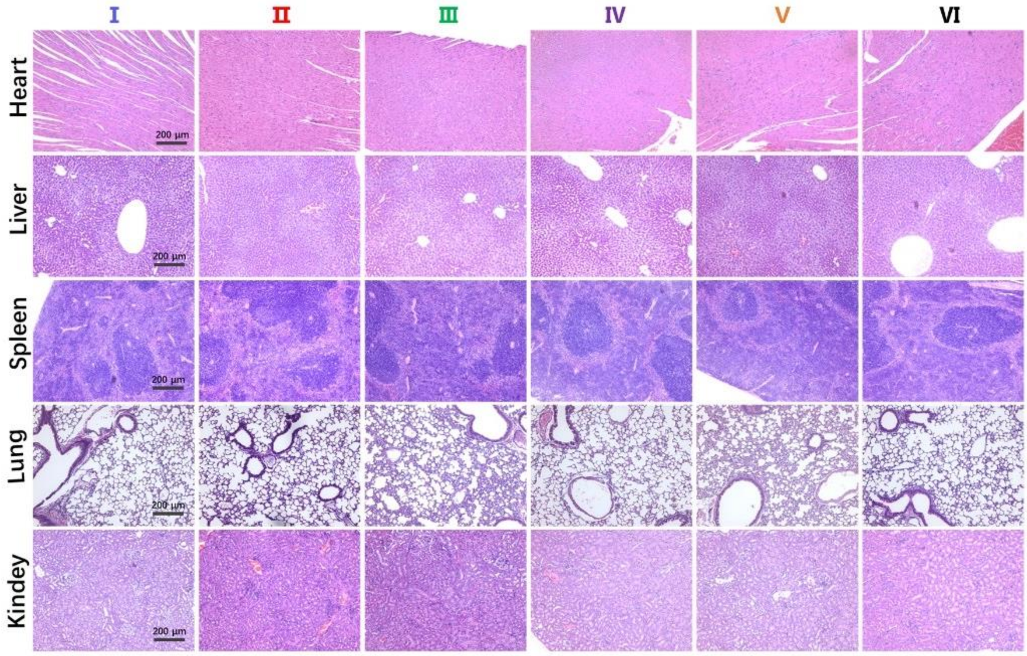

Fig. S17. Hematoxylin and Eosin (H&E) staining assay of B16F10 tumor-bearing mice. H&E staining assay of the main organs (heart, liver, spleen, lung, and kidney) of B16F10 tumor-bearing C57BL/6 mice in different treatment groups. (I: Control, II: PDSA NPs, III: ARV@PDSA (10 mg/kg), IV: ARV@PDSA (3 mg/kg), V: Free ARV-771 (10 mg/kg), VI: Free ARV-771 (3 mg/kg)).
